# Microporous/Macroporous Polycaprolactone Scaffolds for Dental Applications

**DOI:** 10.3390/pharmaceutics15051340

**Published:** 2023-04-26

**Authors:** Tara Shabab, Onur Bas, Bronwin L. Dargaville, Akhilandeshwari Ravichandran, Phong A. Tran, Dietmar W. Hutmacher

**Affiliations:** 1 Faculty of Engineering, School of Mechanical, Medical and Process Engineering, Queensland University of Technology, Brisbane, QLD 4000, Australia; 2 Max Planck Queensland Centre, Brisbane, QLD 4000, Australia; 3 Australian Research Council Training Centre for Multiscale 3D Imaging, Modelling and Manufacturing (M3D Innovation), Queensland University of Technology, Kelvin Grove, QLD 4059, Australia; 4 Australian Research Council Training Centre for Cell and Tissue Engineering Technologies, Queensland University of Technology, Brisbane, QLD 4059, Australia

**Keywords:** architecture, biomateriomics, biomimetic, dental scaffolds, drug delivery, multiphasic

## Abstract

This study leverages the advantages of two fabrication techniques, namely, melt-extrusion-based 3D printing and porogen leaching, to develop multiphasic scaffolds with controllable properties essential for scaffold-guided dental tissue regeneration. Polycaprolactone–salt composites are 3D-printed and salt microparticles within the scaffold struts are leached out, revealing a network of microporosity. Extensive characterization confirms that multiscale scaffolds are highly tuneable in terms of their mechanical properties, degradation kinetics, and surface morphology. It can be seen that the surface roughness of the polycaprolactone scaffolds (9.41 ± 3.01 µm) increases with porogen leaching and the use of larger porogens lead to higher roughness values, reaching 28.75 ± 7.48 µm. Multiscale scaffolds exhibit improved attachment and proliferation of 3T3 fibroblast cells as well as extracellular matrix production, compared with their single-scale counterparts (an approximate 1.5- to 2-fold increase in cellular viability and metabolic activity), suggesting that these structures could potentially lead to improved tissue regeneration due to their favourable and reproducible surface morphology. Finally, various scaffolds designed as a drug delivery device were explored by loading them with the antibiotic drug cefazolin. These studies show that by using a multiphasic scaffold design, a sustained drug release profile can be achieved. The combined results strongly support the further development of these scaffolds for dental tissue regeneration applications.

## 1. Introduction

There is a clinical need for a multifunctional device technology platform that can act as a supportive framework for dental tissue regeneration as well as to act as a reservoir for local drug delivery [1,2,3]. In order to achieve dental tissue regeneration, the design of scaffolds should consider the different phases of wound healing and incorporate various morphological features to guide the complex biological workflow of the host tissue towards regeneration [4]. For example, the different stages of dental bone regeneration exhibit a heterogeneous tissue structure and composition, and thus engineered scaffolds with similar heterogeneity [5], biomechanics and biochemistry [6,7] are desired.

Scaffolds with an interconnected pore network are also critically important, as their high surface area and good mass transport properties improve the permeability and diffusivity of oxygen and nutrients and facilitate the exchange of metabolites [8,9]. One strategy that has been used to work towards this complex goal is to create multiphasic scaffolds that consist of more than one material or have architectures with structural gradients [5]. For example, it has been shown that multiscale scaffolds with pores of different sizes led to enhanced osteointegration and bone growth and an increase in the bone/scaffold interface area compared with their single-phase counterparts [10,11,12].

In addition to this function, scaffolds can be utilized as drug delivery vehicles [13,14]. It is known that contamination of prostheses and implants with endogenous flora during surgery is responsible for most implant-related infections [15,16]. Systemic administration of antibiotics is currently the standard treatment. However, systemic toxicity and dose limitations decrease the treatment efficacy. Hence, a better approach could be the delivery of the drug locally via the device/implant itself [17]. Localized drug delivery minimizes antibiotic dissemination to off-target organs. Similarly, the antibiotic can reach a higher concentration at the site of the implant while minimizing the risks associated with systemic exposure [2,3]. However, the development of multifunctional systems that can be used in both tissue regeneration and drug delivery is a multifaceted problem. Therefore, it is often essential to combine multiple manufacturing techniques along with complex material compositions to address all of the requirements [18,19,20].

This study leverages the advantages of two scaffold fabrication techniques, namely, melt-extrusion-based 3D printing and porogen leaching, to develop multiphasic scaffolds, consisting of an interconnected macro- and microporous network, in order to address the requirements of the complex dental tissue regeneration process.

The goal of biomateriomics is the complete understanding of the biomateriome—a holistic characterization of a complex biomaterial system [21]. The balance of design and function of scaffolds is well recognized, but the biomateriome must encompass a basic characterization of complex multiscale scaffold architectures. The systematic investigation of the design and fabrication of scaffolds has advanced considerably in recent years [22,23,24], along with advancement in the methods required for analysis. Indeed, there are constant updates and refinements of techniques providing new, more accurate means to measure, interpret, quantify and model the relationships between chemistry, structures, design and function of scaffolds. Although the concept of multiphasic scaffolds has been previously explored by various groups, there is a lack of a comprehensive study that thoroughly investigates all the key aspects of such scaffolds and tests their in vitro suitability for tissue engineering applications. In this study, we evaluate the biomateriome of 3D-printed multiscale scaffolds that are loaded with drugs using a wide range of scientific methods and characterisation techniques and provide an often-missed holistic understanding. Additionally, unlike the rest of the literature, the study prioritises the requirements of dental tissue regeneration processes in the design and fabrication of multiphasic scaffolds. The factors investigated here are: the effects of porogen size on the porogen leaching process; printability of the biomaterial and polymer thermal degradation during the manufacturing process; surface properties; mechanical properties; degradation and drug delivery performance of the scaffolds.

## 2. Materials and Methods

**Figure 1:**
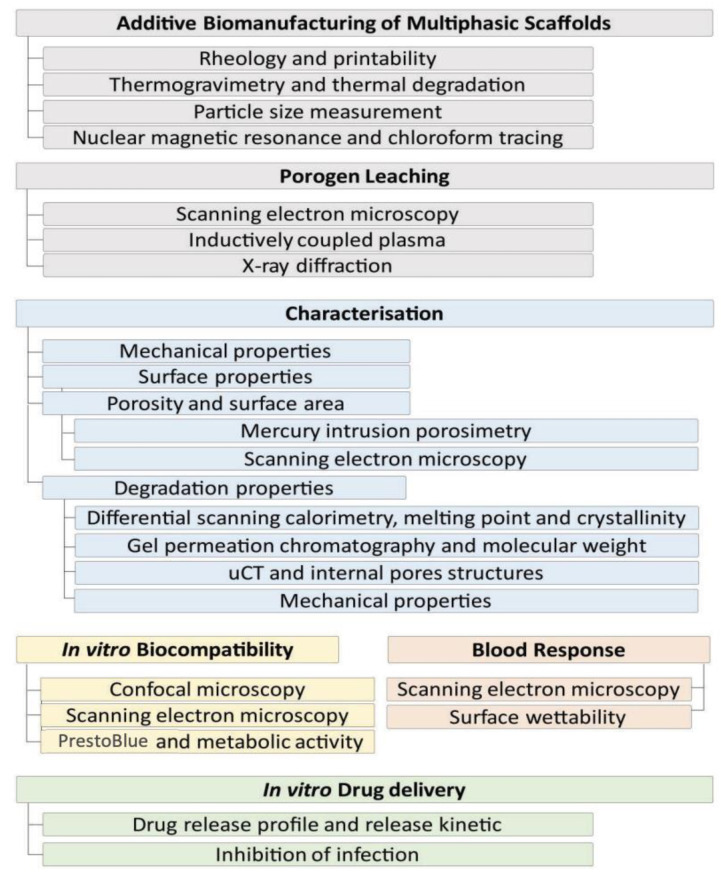
The workflow used in this study, showing the steps involved in manufacturing, scaffold characterisation, in vitro biological response and in vitro drug delivery.

Figure 1 shows a schematic representation of the workflow used in this study.

### 2.1. Pre-Fabrication Studies

#### 2.1.1. Particle Size Characterization

Porogen size distribution was evaluated using dynamic light scattering (DLS) (Malvern Mastersizer 3000 laser light scattering unit, Malvern Instruments Ltd., Malvern, UK). The porogen was dispersed in 100% ethanol and sonicated for 5 min prior to measurement at room temperature (23 °C). The porogen size was also investigated using scanning electron microscopy (SEM) (JEOL 7001F), after the samples were gold-coated at 30 mA for 75 s using Leica EM-SCD005 gold sputter coater (Wetzlar, Germany).

#### 2.1.2. Composite Preparation

Polycaprolactone (PCL)-phosphate-buffered saline (PBS) composite films were prepared using a solvent casting method. The porogen was prepared by grinding PBS tablets (Dulbecco A, Oxoid^TM^) with a mortar and pestle followed by sieving (Sieve No. 100, 170 and 400) (W.S. Tyler, Mentor, OH, USA) to obtain specific particle size ranges. The PCL-based composite films were prepared with two different porogen sizes. The PBS particles with a size of “<30 µm” and “90 µm < porogen < 150 µm” were referred to as P30 and P100, respectively (scaffolds without porogen; hence, no microporosity, were referred to as nonporous (NP)). The porogen was dispersed in chloroform by 3 cycles of stirring for 30 min using a magnetic stirrer, followed by 15 min of sonication. Subsequently, PCL pellets (Capa^tm^ 6800, Perstorp, Malmö, Sweden) were dissolved in the mixture *w*/*w* % (mass of porogen/total mass of PCL and porogen) = 45% and stirred at 100 °C for one hour to obtain homogeneity. Due to the porogen density and high viscosity of the mixture, temperatures below 100 °C could not be used for this step. The PCL–PBS mixture was then cast onto glass surfaces and left for chloroform evaporation at room temperature for 24 h. Subsequently, these films were used as the raw material for the fabrication of porous scaffolds.

#### 2.1.3. Rheology

Rheological experiments were carried out on the PCL–porogen films in order to understand some of the properties relevant to the scaffold manufacturing process. Rheological measurements which included amplitude sweep and shear–viscosity were performed with pure PCL pellets, P30 and P100 composite films using an Anton Parr M302 Rheometer (Anton Paar USA Inc., Ashland, VA, USA) equipped with a cylindrical parallel plate geometry (25 mm diameter plate) at a gap of 1 mm. Amplitude sweep tests were performed at different shear strain rates ranging from 0.01% to 100% at a constant angular frequency of 10 1/s at 150 °C. Shear–viscosity measurements were performed with a shear rate ranging from 0.1 to 100 s^−1^ at 150 °C.

#### 2.1.4. Thermogravimetric Analysis

Thermogravimetric analysis (TGA) analysis was carried out to check the thermal decomposition of the composites (TA instrument Q100, TA Instrument, New Castle, DE, USA). Pure Capa6800 PCL pellets, P30 films and P100 films were heated up to 100 °C (5 °C/min), kept at 100 °C for an hour (in order to simulate the thermal conditions of the 3D-printing process) and then heated up to 1000 °C (5 °C/min) until full decomposition. The experiment was carried out in nitrogen gas with a flow rate of 60 mL/min.

### 2.2. Scaffold Fabrication

#### 2.2.1. Additive Manufacturing

PCL-porogen scaffolds were printed using a melt-extrusion-based additive manufacturing system, Bioextruder [25]. Materials (PCL pellets, P30 films or P100 films) were melted in the material extrusion chamber of the device at a temperature of 180 °C and printed at a translational collector speed of 6.5 mm/s. The voltage and current were set at approximately 7 V and 1.2 A, respectively. Scaffolds were printed through a cylindrical steel industrial needle (G19) with an inner diameter of 0.7 mm. Scaffolds were produced with dimensions of 30 × 30 mm, a laydown pattern of 0/90°, filament distance of 2.4 mm, layer height of 0.51 mm and 80% macroporosity (Figures S1 and S2).

#### 2.2.2. Porogen Leaching

The printed scaffolds were immersed in dilute NaOH solution (0.01 M) for 16 days in an incubator at 37 °C and 200 RPM orbital shaking, in order to leach out the PBS. The scaffolds were vacuum-dried and then weighed. The leaching of the PBS particles through the scaffolds was investigated by SEM. Samples were gold-coated at 30 mA for 75 s (Leica EM-SCD005 gold sputter coater, Wetzlar, Germany) and imaged using a JEOL 7001F microscope. In order to evaluate the degree of PBS leaching and to characterize any PBS residues in the scaffolds, ICP-OES and X-ray diffraction/powder X-ray diffraction (XRD/PXRD) were carried out.

The leaching solutions (2 mL) were collected at different time points (12, 24, 36 and 48 h) and analyzed for cations using a PerkinElmer Optima 8300 dual view ICP-OES. The sample introduction system of the ICP-OES was equipped with a peristaltic pump (0.44 L/min) and a cross-flow nebulizer with argon gas (flow rate of 0.60 L/min). The quartz torch was supported with a 2 mm diameter injector and a cyclonic spray chamber. The plasma was generated by a solid-state 40 MHz RF generator power (1500 Watts). The plasma argon flow rate was 12 L/min and the auxiliary argon gas flow was 0.8 L/min. Emitted wavelengths were measured using a sealed charged coupled device detector. A set of quality control samples were run including analytical water, certified reference materials, and initial check verification standards.

### 2.3. Scaffold Characterization

#### 2.3.1. Chloroform Tracing in Manufactured Scaffolds

Proton nuclear magnetic resonance (^1^H-NMR) was applied to confirm the complete removal of solvent (chloroform) from the cast films and printed scaffolds. P30 leached, P100 leached and NP scaffolds (30 mg) were dissolved in CD_2_Cl_2_ and ^1^H NMR spectra were acquired at 298 K on a Bruker Advance III 600 MHz spectrometer equipped with a 5 mm Broadband Observer probe. ^1^H-NMR chemical shifts are reported in parts per million (ppm) relative to the residual protonated solvent peak.

#### 2.3.2. X-ray Diffraction

XRD was used to measure the crystallite size of PCL-porogen and leached scaffolds. Fixed incidence angle X-ray diffraction patterns were acquired using a Rigaku SmartLab diffractometer (Cu source, 40 kV, 40 mA) operating in parallel beam mode with a Hypix 3000 detector. The incidence angle was fixed to 2°; the scan-axis was 2-Theta. The incident optics consisted of a 5° Soller slit, a 15 mm incident slit and a fixed divergence slit (0.28 mm). This geometry minimizes excessive penetration of the incident beam into the samples at high angles. Receiving optics included a 0.228° parallel plate collimator and 20 mm receiving slits. Patterns were collected using a step size of 0.01° from 5–75° 2θ at 1.2 deg·min^−1^. Phase identification was performed with Highscore Plus (V4, PANalytical) and Jade (V6.8, MDI) using the PDF-4+ databases (2015, International Center for Diffraction Data) (reference patterns: PCL: 00-062-1286, NaCl: 01-070-2509, KCl: 04-006-5938, KH_2_PO_4_: 01-079-0585 and Na_2_HPO_4_.2H_2_O_2_: 00-010-0190). The patterns were modelled using TOPAS (V5, Bruker) via Pawley refinements. The appropriate intensity, peak position and peak shape corrections specific to a fixed incident parallel beam geometry were implemented. An instrument function determined from LaB_6_ (SRM 660C) permitted the estimation of crystallite size, as implemented in TOPAS using the double Voigt approach. A separate peaks phase at c.a. 20° 2θ was used to model the amorphous peak of PCL.

#### 2.3.3. Mechanical Characterization

Uniaxial unconfined compression tests were conducted using an Instron (INSTRON Model 5567 Twin Column, USA) equipped with a 5 kN load-cell. Test specimens were first made using a biopsy punch (8 mm diameter) from a 30 × 30 mm scaffold and compressed at a displacement rate of 0.3 mm/min (n = 6). The compression tests were conducted in air at ambient temperature as well as in PBS at 37 °C. The compressive modulus was calculated from the slope of the initial linear portion of the stress–strain curves of the tested specimens.

#### 2.3.4. Morphological Characterization

Stereomicroscope imaging was used to obtain a macroscopic view of the scaffolds (Stereomicroscope Nikon SMZ 745T). The surface roughness of the scaffolds was evaluated by 2D profilometry (Bruker Dektak Stylus Profiler, Billerica, MA, USA). The measurements were carried out on 10 mm lengths of 9 different random struts and were analyzed by Bruker Vision64 software. The stylus tip radius was 2 µm. The 3D surface topography was evaluated using 3D optical profilometry (Zeta 300 3D optical profiler, KLA Instruments, Milpitas, CA, USA). SEM was carried out as explained previously. Microtomography (micro-CT) was carried out to provide information on the scaffold 3D morphology (microCT50, Scanco Medical, Bruettisellen, Switzerland—2 µm voxel size, 45 kV, 133 μA). The surface profile was acquired by a stylus method with a small mechanical contacting force. The lower threshold was set at 40 and the upper threshold was set at 1000 to isolate the PCL scaffold from the background for the evaluation of different morphological parameters.

#### 2.3.5. Accelerated Degradation

The accelerated hydrolytic degradation was studied by submerging the scaffolds in sodium hydroxide (NaOH) aqueous solution. Each scaffold (25–30 mg) was punched with an 8 mm biopsy punch and samples were submerged in individual tubes containing 1 mL NaOH (2M, pH = 13.0). The tubes were maintained at 37 °C in an orbital shaker at 200 rpm. PCL scaffolds were removed after 12, 24, 36 and 48 h, dried in a vacuum oven at 30 °C for 12 h and weighed before further analysis. The percentage of mass loss was calculated using the following equation:

% Mass loss = (W_o_ − W_d_)/W_o_ × 100

where W_o_ is the weight before degradation and W_d_ is the weight after the degradation.

The pH of the solutions was measured at each time point. Filament thickness before and during degradation was measured by ImageJ software on SEM micrographs imaged by Tescan MIRA3.

#### 2.3.6. Mechanical Properties after Degradation

In order to investigate the effect of microporosity on the mechanical behaviour of the scaffolds during accelerated hydrolytic degradation, mechanical compression tests were conducted as described above.

#### 2.3.7. Differential Scanning Calorimetry

Differential scanning calorimetry (DSC) was used to study the thermal properties and crystallinity of the degraded PCL scaffolds. DSC was performed on a TA instrument Chimaera Q100 (USA) and analyzed by Universal Analysis 2000 software. In total, 3–4 mg of each sample was tested. The samples were first cooled down to −60 °C at a rate of 10 °C/min and then they were heated to 200 °C at a rate of 10 °C/min. Melting temperatures (T_m_) were obtained at the peak of the melting endotherms. Melting enthalpies (ΔH_m_) were obtained from the areas under the peaks. The degree of crystallinity (% X_c_) was determined as:

% X_c_ = (ΔH_m_/ΔH^0^_m_) × 100;

where ΔH_m_ is the measured experimental heat of fusion normalized to the mass of the PCL, and ΔH^0^_m_ is the specific heat of fusion of 100% crystalline PCL taken as 139.5 J/g [26,27].

#### 2.3.8. Gel Permeation Chromatography

Gel permeation chromatography (GPC) was carried out to study the molecular weight reduction of PCL scaffolds during degradation. All samples were dissolved in tetrahydrofuran (THF) and passed over 0.22 µm PTFE membrane filters. GPC measurements were performed on a PSS SECurity2 system consisting of a PSS SECurity Degasser, PSS SECurity TCC6000 Column Oven (35 °C), PSS SDV Column Set (8 × 150 mm 5 µm precolumn, 8 × 300 mm 5 µm analytical columns, 100,000 Å, 1000 Å, and 100 Å) and an Agilent 1260 Infinity Isocratic Pump, Agilent 1260 Infinity Standard Autosampler, Agilent 1260 Infinity Diode Array and Multiple Wavelength Detector (A: 254 nm, B: 360 nm), Agilent 1260 Infinity Refractive Index Detector (35 °C). HPLC grade THF, stabilized with BHT, was used as eluent at a flow rate of 1 mL/min. Poly (methyl methacrylate) (M_n_: 202 g·mol^−1^ to 2.2 × 10^6^ g·mol^−1^) standards (PSS ReadyCal) were used as calibrants. Molecular weight and dispersity analyses were performed in PSS WinGPC UniChrom software (version 8.2).

#### 2.3.9. Micro-CT Imaging

Micro-CT imaging was carried out as explained previously to provide information on the 3D morphology of the scaffold and the pores. Changes in pore internal structure and interconnectivity of the pore microporosity were investigated by micro-CT after 48 h of accelerated degradation. Two-dimensional porosity before and after degradation was calculated using ImageJ software (version 1.53) based on micro-CT 2D images. Three-dimensional microporosity before and after degradation was calculated using micro-CT software.

#### 2.3.10. Mercury Intrusion Porosimetry

Mercury intrusion porosimetry (MIP) was carried out to measure the surface area and permeability of the porous scaffolds (Micromeritics PoreSizer 9320). The penetrometer chamber containing the samples (roughly 0.21 g) was degassed, then filled with mercury and the pressure increased to 60,000 psi (414 MPa) to force mercury into the pores.

#### 2.3.11. Porous Film Surface Characterization

The hydrophilicity of PCL scaffolds was evaluated using contact angle measurement. Images of contact angle measurement were recorded and analyzed using an FTA 200 analyzer (First Ten Angstroms, Newark, CA, USA). In total, 20 µL of Milli-Q water was dropped on the surface of the samples and the contact angle was measured after 5 s.

### 2.4. Biological Characterization

In this study, the effect of the scaffold surface on fibroblast cell response was investigated. SEM, confocal microscopy and PrestoBlue assay were used to investigate the effect of surface topography and roughness on fibroblast cell adhesion and proliferation, and the details are described below.

#### 2.4.1. Plasma Treatment

Prior to cell culture, the scaffolds were plasma treated (gas flow mixer, PlasmaFlo, PDC-FMG-2, and Plasma cleaner, PDC-002-HP (Harrick Plasma, Ithaca, NY, USA). Coupled plasma (argon and oxygen) treatments were carried out at low radiofrequency discharge for 120 s on each side of the scaffold.

#### 2.4.2. Fibroblast Cell Culture

The plasma-treated scaffolds were sterilized in 80% ethanol (*v*/*v*) for 30 min and dried under aseptic conditions. 3T3 fibroblast cells (3T3-Swiss albino, ATCC^®^ CCL-92™) were cultured in flasks at a seeding density of 4000 cells/cm^2^ and maintained in Dulbecco’s modified Eagle’s medium (DMEM) (Gibco, Thermofisher Scientific, Waltham, MA, USA) supplemented with 10% fetal bovine serum (FBS) (Gibco, Thermofisher Scientific) and 1% penicillin and streptomycin (GIBCO, Thermofisher Scientific). Cells were trypsinized at 70% confluence and each scaffold was seeded with 0.12 million cells. Cell media suspension (20 µL) was seeded on one side of the scaffold (Size: 8 mm × 8 mm) and cells were allowed to attach to the scaffold for 30 min at 37 °C and 5% CO_2_. This was repeated on the other side of the scaffold followed by a 30 min incubation, before the addition of 2 mL of culture media per scaffold in a 24-well plate. Here, the day of cell seeding has been assigned as Day 0. The cellular scaffolds were transferred to a new 24-well plate containing fresh media on Day 3.

#### 2.4.3. Cell Morphology Analysis Using Scanning Electron Microscopy

The seeded scaffolds were collected on Day 3 and Day 10 and washed twice with PBS and fixed in 3% glutaraldehyde diluted in cacodylate buffer. The fixed samples were kept at 4 °C and after a few days they were washed twice with PBS and dehydrated using a series of ethanol solutions in water (from 40 to 100% (*v*/*v*)), each time for 30 min followed by hexamethyldisilazane (twice for 30 min). The samples were left inside the hood at room temperature for 24 h prior to imaging (Tescan MIRA3), as described previously.

#### 2.4.4. Quantitative Metabolic Activity by PrestoBlue Assay

PrestoBlue™ cell viability assay (Invitrogen, Thermofisher Scientific) was performed on cell-seeded scaffolds in a 24-well plate. Briefly, 100 µL of PrestoBlue™ reagent (diluted 1:100 (*v*/*v*) in culture media) was added to the scaffolds and incubated for 4.5 h at 37 °C and 5% CO_2_. Cellular viability was assessed by measuring fluorescence at ex/em 540/590 nm using a microplate reader (POLARstar OPTIMA, BMG Labtech, Mornington, Australia) relative to blank wells (PrestoBlue™ reagent diluted in media without scaffolds). In total, 2 replicate measurements were conducted from 4 different samples in each group (NP, P30 and P100) (8 total, 3 replicates for controls/blank). Results were reported as mean ± SD.

#### 2.4.5. Immunofluorescence Staining and Confocal Microscopy

Cell response was monitored on Day 3 and Day 10 by a confocal microscope (SP5, Leica). Cell-seeded scaffolds were gently washed twice with PBS and then fixed with 4% paraformaldehyde (PFA) in PBS at room temperature (RT) for 30 min. The fixed scaffolds were kept in the fridge at 4 °C for a few days before staining. To visualize the focal adhesion established by cells on the PCL scaffolds, primary antibody anti-FAK (ab39967, Abcam) was used. Firstly, the scaffolds were washed with PBS and permeabilized with 2% bovine serum albumin (BSA) (Sigma Aldrich, St. Louis, MI, USA) containing 0.2% Triton X-100 (Merck) in PBS for 1 h at room temperature on a plate shaker. This was followed by incubation with 2% BSA in PBS for 2 h at room temperature on a plate shaker to block non-specific antibody binding. The samples were then incubated with the primary antibody solution for FAK, (ab39967 FAK, diluted 1:100 in 1% BSA) overnight at 4 °C. Subsequently, the samples were rinsed in PBS for 9 h and then incubated with the Goat anti-rabbit 488 (A11008, Invitrogen, Thermofisher Scientific, 1:200), DAPI (D1306, Invitrogen, Thermofisher Scientific, 1:1000) and Rhodamine phalloidin 568 (R415, Invitrogen, Thermofisher Scientific, 1:250) for 45 min at room temperature on a plate shaker. Finally, the samples were washed for 5 h in PBS and imaged with the confocal microscope.

#### 2.4.6. Surface and Architecture Interaction with Blood

Polymer–blood interaction was performed on PCL films (NP, P30 and P100). PCL films (6 mm × 6 mm) were sterilized in 70% (*v*/*v*) filtered ethanol. Whole sheep blood was centrifuged at 4000 rpm for 10 min to collect the plasma. Then, the plasma was centrifuged at 5000 rpm for 10 min and the bottom third of the tube was collected as platelet-rich-plasma (PRP). To characterize the interaction between the blood and PCL, the films were incubated in whole sheep blood and PRP for an hour at 37 °C in a 12-rpm shaker incubator. Plasma clotting and whole blood clotting were performed on the films to investigate the difference in the clotting and fibrin network formation between the groups. Clotting was initiated by recalcifying sheep whole blood and sheep plasma with CaCl_2_ solution (the final molarity of 12.5 mM). Subsequently, 50 µL of the whole blood clot and plasma clot was added immediately to the films and incubated for an hour at 37 °C in a 12 rpm shaker incubator. Incubated samples (whole blood, PRP, whole blood clot and plasma clot) were washed with PBS (×2), fixed in 3% glutaraldehyde diluted in cacodylate buffer for an hour at 4 °C, washed with PBS and dehydrated using a series of ethanol solutions in water (50%, 70%, 90% and 100% (*v*/*v*)) followed by hexamethyldisilazane. The samples were gold coated (Leica EM-SCD005 gold sputter coater, Wetzlar, Germany) for SEM imaging (Tescan MIRA3, Tescan, Brno, Czech Republic). Fibrin fibre diameter was measured from SEM micrographs, using ImageJ software. In total, 60 random fibres in 803 µm^2^ area on SEM micrographs in each group were analyzed. RBC and platelet counting were performed using ImageJ software on 32 µm^2^ areas of SEM micrographs.

### 2.5. In Vitro Drug Delivery

Cefazolin (PF-00345346, Pfizer, New York, NY, USA), a water-soluble and negatively charged molecule [28], available as its sodium salt, was used as an antibiotic model for the drug delivery experiment. Scaffolds (6 mm × 6 mm) were first sterilized in 70% ethanol (*v*/*v*). Cefazolin was dissolved in 90% ethanol and added to microcentrifuge tubes containing sterile scaffolds according to 2 doses: high dose (HD = 0.6 mg/mL) and low dose (LD = 0.06 mg/mL). Scaffolds were kept under vacuum for 20 min to induce the drug into the pores and then left to dry overnight inside a sterile biological cabinet. The total drug amount loaded on the scaffolds was 12 µg in low dose and 120 µg in high dose scaffolds. Drug-loaded scaffolds were transferred to fresh tubes and 0.2 mL PBS was added to the tubes to perform the release study in sink conditions, to ensure a continual concentration gradient for release while maintaining the presence of the drug at a detectable level. The samples were placed in a shaker incubator at 200 rpm and 37 °C. At each time point, 50 µL of the supernatant containing the released compound was collected and transferred to an ultraviolet-visible transparent plate. The solution inside each tube was refreshed. The concentration of drug loss from the loading solutions and the collected drug from the release solutions were monitored by measuring the absorbance of the drug molecule at 272 nm [29] using a microplate spectrophotometer (Bio-Rad, Hercules, CA, USA). The amount of drug loss in the tubes during the drug loading process and drug release from the scaffolds were calculated using a standard curve.

^1^H-NMR was utilised in order to determine whether any drug remained within the scaffolds after the release experiment. Approximately, 40 mg of drug-released scaffolds (NP, P30, P100) were dissolved in 0.5 mL of CDCl_3_ in a micro-glass vial. Afterwards, 0.5 mL of D_2_O was added and mixed well by shaking the vial, followed by standing for an hour to allow phase separation (one phase D_2_O and cefazolin, and the other phase CDCl_3_ and PCL). ^1^H NMR spectra of the D_2_O phase were acquired at 298 K on a Bruker Advance III 600 MHz spectrometer equipped with a 5mm BBO probe. ^1^H-NMR chemical shifts are reported in parts per million (ppm) relative to the residual protonated solvent peak.

Moreover, the drug-released scaffolds were etched by NaOH (2M; 200 µL in each tube) and after 2 h in a shaker incubator (200 rpm and 37 °C), 50 µL of the supernatant was collected and transferred to an ultraviolet-visible transparent plate and the absorbance was measured at 272 nm [29] using a microplate spectrophotometer (Bio-Rad, US).

The antimicrobial properties of the drug-loaded scaffolds were evaluated using the agar diffusion method against *Staphylococcus aureus* (ATCC 29213). *S. aureus* was cultured on Trypticase Soy Agar and incubated overnight at 37 °C. After overnight growth, 0.5 McFarland bacterial solution was prepared and 100 µL of the solution was spread on Mueller-Hinton agar (MHA) plates. Cefazolin-loaded scaffolds were placed on the plates. Non-drug scaffolds were used as a negative control. Oxoid^TM^ cefazolin antimicrobial susceptibility disks (30 µg) were used as a positive control. The plates were incubated for 24 h at 37 °C and the inhibition zones were measured.

### 2.6. Statistical Analysis

Data are presented as mean ± standard deviation (SD). The significant differences (*p* < 0.05) between experimental groups were determined by a one-way ANOVA test, followed by a Tukey’s multiple comparisons test using GraphPad Prism 8 analysis software. *, ** and *** in figures indicate *p* < 0.033, *p* < 0.002 and *p* < 0.001, respectively.

## 3. Results and Discussion

### 3.1. Scaffold Design and Manufacturing

As biocompatibility is one of the most important aspects in the design of implantable devices [30], including tissue-engineered products, polycaprolactone (PCL) was preferred in the fabrication of the scaffolds due to its proven track record in the biomedical applications. PCL, a semi-crystalline biodegradable polymer belonging to the aliphatic polyester family, has shown great potential in medical applications, tissue engineering, and drug delivery, and has already been used in several medical devices approved by the FDA [31,32]. Moreover, PCL has a low melting point (~60 °C) and good solubility in organic solvents; therefore, is easy to process, which makes this polyester an outstanding candidate for 3D-printed scaffolds [33].

**Figure 2:**
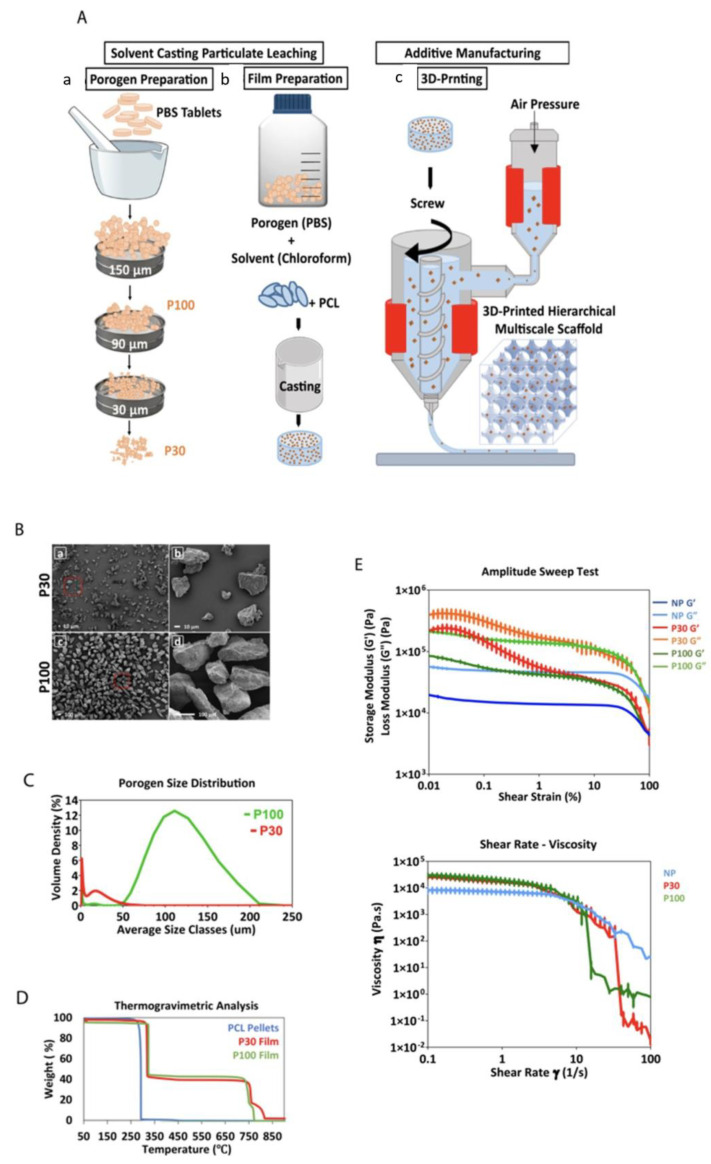
(**A**) Schematic presentation of the manufacturing of PCL multiphasic scaffolds. (**B**) SEM of the porogen particles, (**a**,**b**) different magnifications of P30 porogen, (**c**,**d**) different magnifications of P100 porogen. (**C**) Porogen size distribution determined by dynamic light scattering (n = 5). (**D**) TGA analysis showing the decomposition temperature of the materials. (**E**) Rheological properties of the materials (PCL pellets, P30, and P100 films) (n = 3).

In this study, scaffolds were manufactured by combining a porogen leaching process, referred to as solvent casting/particle leaching (SCPL), and additive manufacturing (AM) methods (Figure 2A). SCPL is a popular, simple and low-cost technique used for the fabrication of porous scaffolds [34,35]. The pore characteristics of the scaffolds in this process can be controlled by the size, shape, and concentration of the porogen [36]. However, casting large scaffolds is not possible with this technique since the porogen will not leach out through thickly cast structures. However, combining the microporosity achieved via SCPL with the microporosity imparted by additive manufacturing (AM) technologies, allows the possibility of forming a fully interconnected pore network based on a patient-specific computer-aided design (CAD) model in a reproducible manner [37]. Moreover, AM systems are capable of printing composite polymeric scaffolds containing various inorganic particles in order to improve mechanical and biological properties [38]. The printer used in this study (Bioextruder) combines pneumatic and screw-driven systems; therefore, it has a high extrusion power that can process highly viscous polymers [39] (Figure S2). Importantly, and in contrast to fused deposition modelling, Bioextruder is an additive manufacturing system, consisting of a head-heated liquefier, that can process polymers and their composites in various forms such as filament, pellets, powder, etc. [40]. The movement of the translational collector is governed by G-codes, which can be programmed to obtain the desired scaffold macro-architectures [41].

Both surface roughness and microporosity increase surface area and enhance cell attachment [42,43]. Therefore, P30 (porogen < 38 µm) and P100 (90 µm < porogen < 150 µm) were chosen as two porogen size groups for SCPL processing in this study. The macroporosity (>1.5 mm) µm was introduced by additive manufacturing. Phosphate buffered saline (PBS) (solid particles) was used as the porogen in this study. PCL/PBS composites were first prepared by solution casting of the mixture. The optimum PBS to PCL ratio in the composite was found to be 45% (*w*/*w*). The pilot studies showed that PCL with higher PBS porogen content was unprintable due to its very high viscosity. Lower porogen ratio led to a high proportion of closed pores, which did not allow enough micropore interconnectivity to facilitate full salt leaching. Additionally, the use of porogen > 150 µm led to the clogging of the G19 needle used in this study. It was identified that P100 was more difficult to extrude in comparison to P30. Such optimisation studies are essential to ensure easy and reproducible scaffold manufacturing conditions as well as to avoid the risk of entrapped salt residues, which can negatively affect the biological performance of the resulting scaffolds. Additional studies are recommended if filament diameter is altered in the scaffolds as this can lead to different leaching behaviours.

Figure 2B,C show the scanning electron microscopy (SEM) micrographs and the size distribution graphs of both P30 and P100 porogen particles. The SEM micrographs confirmed that the size of the P100 porogen is within the targeted range. Although partially clumped microparticles were found in the SEM images of P30 group (porogen only) (Figure 2B), the sonication of the particle suspension before film casting resolved the issue and led to uniform particle distribution within the polymer. The dynamic light scattering (DLS) results showed that 90% of the P30 and P100 particles were less than 24.04 ± 4.1 and 160.0 ± 1.41 µm, respectively (Figure 2C). It is important to underline that uniform particle distribution is important as it prevents the emergence of localised weak points within the scaffolds, which can lead to premature mechanical failures under loading.

The exposure of aliphatic polyesters to high temperatures creates a risk of thermal decomposition of the molecular structure [44]. Thermogravimetric analysis (TGA) results showed that the decomposition temperature of the P30 and P100 films was approximately 321 °C, whereas the thermal decomposition of pure PCL pellets occurred around 290 °C (Figure 2D). At approximately 800 °C, the PBS started to vaporize, and the weight decreased. TGA analysis also showed that after maintaining PCL pellets, P30 and P100 films at 100 °C for an hour, in order to mimic the thermal conditions of the printing process, only weight reduction of approximately 0.12, 0.04, and 0.05% was determined, respectively. Overall, these results suggest that PCL and the composite materials would exhibit very good thermal stability at the printing temperature used in this study. During film preparation, chloroform was used as the solvent for PCL. The proton nuclear magnetic resonance (^1^H-NMR) spectrum of PCL confirmed complete chloroform evaporation after casting, which is important since residual chloroform would potentially be a biological hazard in the resulting scaffolds (Figure S13).

Composite printability is dependent on the material’s rheological properties [45]. The type of porogen and its size affect the rheological properties of composites. For extrusion-based printing, materials should undergo non-Newtonian shear-thinning behaviour to facilitate the extrusion of the material through the nozzle. Upon deposition, higher storage modulus and zero-shear viscosity of the composite at low shear rates help maintain the fidelity of the printed filamentary [46,47]. The viscosity of shear-thinning materials is shear-rate-dependent and decreases with an increasing shear rate (Figure 2E).

The viscoelasticity of PCL and PCL–porogen composites was characterized to evaluate their suitability for extrusion-based printing. The rheological studies in Figure 2E showed that loss moduli (G″) were always greater than storage moduli (G′) over the tested temperature range. In particular, amplitude sweep measurements showed a linear viscoelastic region (LVR) for PCL pellets. Under lower shear strain, G″ and G′ stayed relatively constant and independent of the shear strain. This linear behaviour suggested that the internal friction of PCL pellets was independent of the strain rate. However, this LVR was not conserved for PCL–porogen composites. At low strain and the same temperature, composites with porogen microparticles exhibited more solid-like properties when compared with PCL pellets. With a shear strain approaching 100%, the values of G′ and G″ of porogen–PCL composites decreased and reached those of PCL pellets, indicating similar flow properties for both groups at high shear strains.

Shear–viscosity curves of all samples showed non-Newtonian and shear-thinning behaviour. The results confirmed that the material viscosity should decrease in response to the high shear rates in the extruder nozzle during 3D printing, allowing it to flow freely through the nozzle. Overall, it was identified that screw-driven extrusion systems are suitable for additive manufacturing of PCL–porogen composites due to the involvement of high shear forces. Particle size measurement, TGA and rheology analysis all predicted that the manufacture of the desired polymer–salt composite scaffolds would be successful.

**Figure 3:**
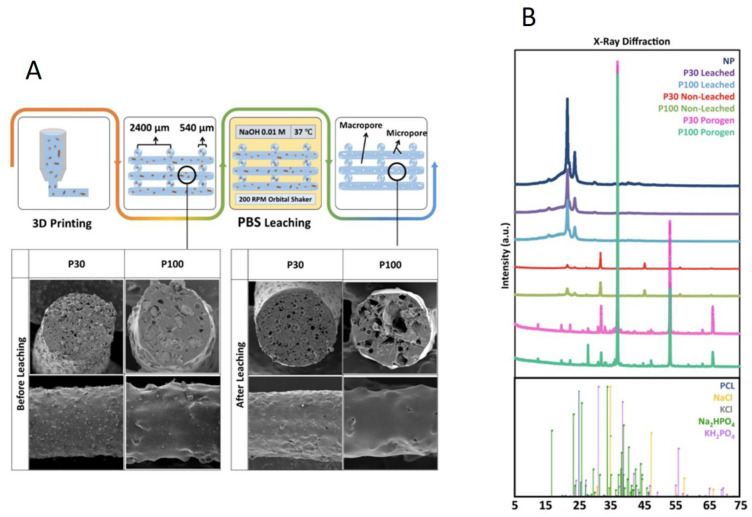
(**A**) Composite leaching process and SEM micrographs. (**B**) X-ray diffraction analysis of the scaffolds, confirming salt leaching (n = 3).

Salt leaching by immersion of the printed scaffolds in 0.01 M NaOH solution for 16 days resulted in complete PBS removal, confirmed by the observation of a 45% weight loss following the process (Figure S12A). The micrographs in Figure 3A and Figure S11 show the porogen inside the scaffolds before leaching, as well as the microstructure of the pores after salt removal. After 16 days of submerging similar scaffolds without porogen (non-porous (NP) scaffolds) in the same solution, no effect was seen in the micrographs. This indicates that this concentration of NaOH did not have a visible impact on the surface morphology of the scaffolds and did not initiate any detectable degradation (Figure S9). Inductively coupled plasma optical emission spectrometry (ICP-OES) analysis of the main elements present in PBS (Na, K, Cl and P) demonstrated salt leaching over the 16 days (Figure S12B). X-ray diffraction/powder X-ray diffraction (XRD/PXRD) analysis also supported the ICP-OES results, confirming salt leaching through the scaffolds (Figure 3B). As expected, porogen–PCL samples showed additional peaks corresponding to PBS powder, compared to the PCL-alone group. KH_2_PO_4_ (potassium dihydrogen phosphate), Na_2_HPO_4_.2H_2_O (disodium hydrogen phosphate dihydrate), KCl (potassium chloride) and NaCl (sodium chloride) are the main components of PBS that are detected by PXRD. KH_2_PO_4_ can be identified at 2θ angles around 32°, 37° and 53°. Na_2_HPO_4_ can be identified at 2θ angles around 32° and 37°. The characteristic peaks at 2θ angles 32° and 67° correspond to the reflection of the NaCl and KCl, respectively. XRD analysis confirmed that the peaks for these species disappeared in leached samples. In summary, weight loss measurement, SEM, ICP-OES and XRD analysis all confirmed complete salt leaching. Production of the microarchitecture in this controlled manner can be used as a tool to tailor the manufacture of scaffolds with desired microporosity and pore interconnectivity for different applications.

By combining the different manufacturing techniques used in this study and by modifying the porogen particle size, concentration and printing parameters, it will be possible to tailor future scaffolds for an array of specific tissue engineering applications. Different porogen sizes can be used to introduce microporosity which can be a tool to design and manufacture tissue and application-specific scaffolds with optimized features, such as interconnectivity, mechanical, degradation and surface properties.

### 3.2. Mechanical and Surface Characterization

The multiscale concept is inspired by the hierarchical structure of biological materials, such as bone [48]. Ideal tissue scaffolds should be highly porous to provide a structure with channels for tissue growth and nutrition delivery while maintaining strength to support the mechanical load [49,50]. Improving the porosity of the scaffolds for efficient mass transport and faster tissue regeneration, without compromising their mechanical properties, is a challenge [51]. Cell attachment and proliferation are also dependant on cell mechanoresponse, which is related to scaffold rigidity [52]. Activation of mechanotransduction pathways modulates biochemical signalling pathways [53] and protein expression, which affect cell proliferation [54]. Biomechanically stable scaffolds are, therefore, needed to protect the newly formed tissue from excessive stresses and strains. In parallel with these requirements, the scaffold should biomechanically conform to its surroundings [55].

Macroporosity and microporosity in the scaffolds can be used as tools to achieve the desired biomechanical properties. In bone tissue engineering, external and internal fixation systems are applied to take the majority of the load-bearing forces. Hence, biodegradable bone scaffolds do not necessarily need to provide complete mechanical support. However, their stiffness and strength should be sufficient and optimized to support forces experienced by the host tissue [20].

**Figure 4:**
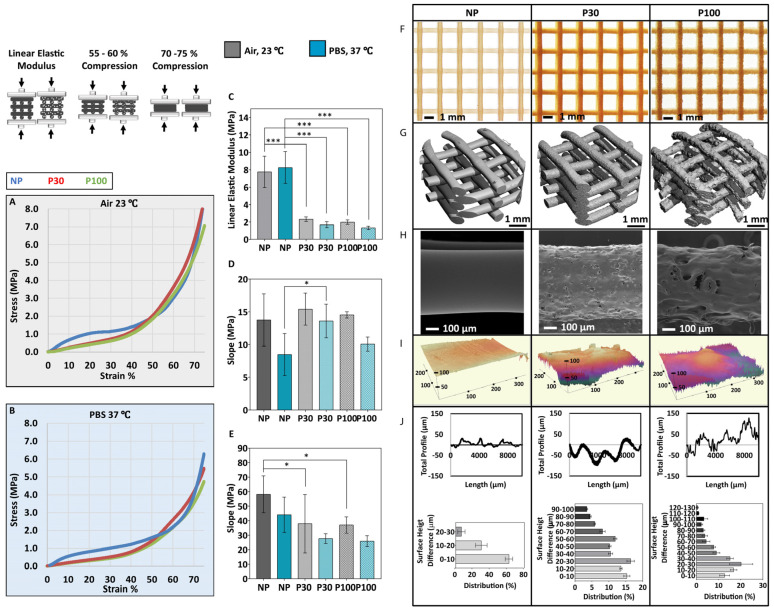
Mechanical compression testing performed in air (**A**) and in PBS (**B**) (n = 6); (**C**) Elastic modulus of the scaffolds; (**D**) Slope of the curve 55–60% compression; (**E**) Slope of the curve 70–75% compression; (**F**) Stereomicroscope imaging; (**G**) the micro-CT 3D reconstruction shows the general topography of the scaffolds; (**H**) SEM and (**I**) 3D profiles show the topography of the scaffolds; (**J**) Scaffold surface roughness (n = 9). Data are presented as mean ± SD. * and *** indicate *p* < 0.033 and *p* < 0.001, respectively.

The influence of porosity on the mechanical performance of the scaffolds was investigated through a series of mechanical tests performed in PBS at 37 °C and in the air at ambient temperature (Figure 4A–E). The compression curve is characterized by three different regions. The first stage corresponds to the linear elastic response of the scaffold. The linear elastic modulus was calculated based on the slope of the linear region of the stress–strain curves (up to 15% compression in porous and 5% compression in nonporous scaffolds) [49] (Figure 4C). The second stage corresponds to the progressive collapse of the micropore structure of the filaments. Figure 4D shows the slope of the compressive curve during this second phase, between 55 and 60% compression. In the third stage, progressive densification of the structure was observed due to the total collapse of the macropores of the scaffolds. This occurs above 70% compression and the slope of this part of the curve (70–75% compression) is shown in Figure 4E.

The elastic modulus of tested samples in the air at ambient conditions showed a significant difference among all the groups (NP (7.76 ± 1.80 MPa), P30 (2.32 ± 0.26 MPa) and P100 (1.98 ± 0.25 MPa)). Similarly, significantly different elastic modulus values were calculated for all the groups when tested in PBS at 37 °C (NP (8.25 ± 1.84 MPa), P30 (1.68 ± 0.34 MPa) and P100 (1.33 ± 0.20 MPa)). The elastic moduli of different human tissues range from 11 Pa to 20 GPa [49]. The elastic modulus range of bone tissue is between 0.1 and 40 GPa, depending on the anatomical site and the type of the bone, whether it is cortical or cancellous [56,57,58]. The elastic modulus range of soft tissue is between 0.5 MPa and 100 MPa, depending on the type of the tissue, whether it is cartilage, skin, breast, neuron, etc. [59]. The scaffolds manufactured in this study are mechanically suitable for soft tissue implantation and could also be applied to bone tissue scaffolding, with the help of plates to achieve the required stiffness [60].

The implant surface properties affect cell response [61] and the tissue regeneration process [62]. Topography influences the tribological properties of the scaffolds [63,64]. Moreover, the microtopography of the scaffolds can affect cell adhesion [65], in a cell-type-dependent manner. For example, rougher surfaces favour cell attachment, proliferation and differentiation of osteoblasts [66,67]. The macroscopic and microscopic view of the scaffolds is shown by stereomicroscope imaging (Figure 4F) and SEM imaging (Figure 4H). Figure 4G shows micro-CT 3D reconstruction and topography of the scaffolds. The 3D profile showed the higher roughness of the porous scaffolds in comparison to nonporous (NP) scaffolds (Figure 4I), where surface roughness increased with increasing porogen size. The surface roughness increased from 9.417 ± 3.01 µm in NP scaffolds to 19.71 ± 6.27 µm in P30 and 28.75 ± 7.48 µm in P100 scaffolds (Figure 4J). The suitability of surface parameters, topographical features (grooves, pillars, wells, pits, pyramidal-shapes and cavities with curved surfaces) and the size of the features are expected to vary widely for different cell types and different applications, and materials and would need to be evaluated on a case-by-case basis [53]. For example, in a previous study, the height and spacing of the pillars influenced the fibroblast cell morphology and spreading and by decreasing the pillar heights the cells showed morphologies similar to cells cultured on flat surfaces [68,69]. In another study, fibroblast and breast cancer cells were cultured on curved surfaces. Results showed that the fibroblast cells were stretched, whereas the breast cancer cells adopted the shape of the curved surfaces [70,71,72]. Moreover, treating breast cancer cells with anticancer drugs led to stretched morphology similar to fibroblast cells [73].

### 3.3. Scaffold Degradation

The scaffold structure guides the development of new tissue and is responsible for mechanical support at the site of the implant until the new tissue forms. After the tissue remodels and matures, the scaffold should undergo degradation so that it can be replaced with natural tissue [67]. Thus, the rate of resorption of the implant is an important parameter in the design of tissue engineering scaffolds [74]. Depending on the type of implant and tissue growth, different degradation rates are considered suitable [75]. Moreover, the combination of different microporosity in different sections of one scaffold can be beneficial where the tissue remodelling is variable in different parts of the organ or tissue.

Under physiological conditions, PCL undergoes non-enzymatic hydrolytic degradation by bulk erosion due to the permeation of water into the pores of the scaffold and into the polymer molecular network itself. Degradation occurs via cleavage of the ester linkages, resulting in a corresponding reduction in molecular weight [9]. Consequently, as the polymer chains are cleaved into smaller oligomers, carboxyl and hydroxyl end-groups are generated. Upon complete degradation, these oligomers become monomeric hexanoic acids. Under physiological conditions, the complete degradation of PCL is very slow [76] (>2 years). In the current study, to be able to investigate the morphological and chemical changes of scaffolds undergoing similar hydrolytic degradation in a more experimentally acceptable time frame (48 h), 2M NaOH solution was used to accelerate the process [77], as the hydrolysis of the ester bonds in PCL is catalyzed in the presence of a base (Figure S14A).

In addition to the inherent properties of the materials used, the porosity of the scaffolds influences the degradation kinetics. As porosity increases, the total surface area per unit weight of material increases, which generally leads to faster degradation [78,79]. SEM, differential scanning calorimetry (DSC), gel permeation chromatography (GPC), micro-CT and mechanical tests were performed for the characterization of degraded samples.

The pH remained the same (approximately 13.0) during the accelerated degradation study performed in 2M NaOH (48 h). NP scaffolds did not show a significant weight loss, even after 48 h. This is due to their low surface area-to-volume ratio that minimises the surface area exposed to 2M NaOH. P30 showed a greater weight loss in comparison to P100 (Figure S14C). P30 scaffolds lost 9.11 ± 1.26%, 25.40 ± 6.02% and 36.30 ± 2.15% of their weight after 24, 36 and 48 h, respectively, whereas the weight loss of P100 scaffolds was found to be 6.69 ± 2.25%, 16.58 ± 3.19% and 25.68 ± 3.88% after 24, 36 and 48 h, respectively. P30 scaffolds also exhibited the largest reduction in filament diameter (from 595.00 ± 5.00 µm at 0 h to 390.00 ± 62.23 µm at 48 h) (Figure S14D). P100 scaffolds showed a significant decrease in filament diameter from 692.50 ± 65.14 µm at 0 h to 556.50 ± 0.71 µm after 36 h. These results could be attributed to the greater surface area of the P30 samples (see later discussion of Micro-CT and Mercury intrusion porosimetry), leading to enhanced penetration of the degradation medium and consequently faster degradation.

**Figure 5:**
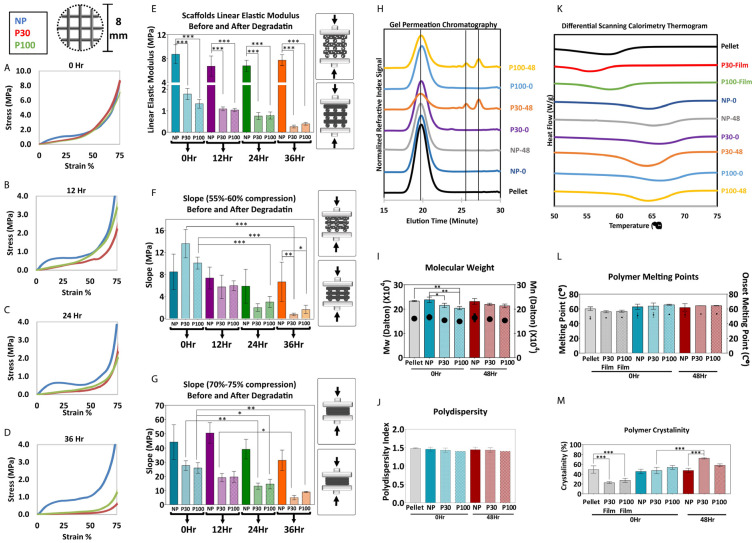
(**A**–**G**) Mechanical compression test during degradation (n = 5). (**H**–**J**) GPC shows molecular weight reduction and an increase in polydispersity (n = 3). (**K**–**M**) DSC shows the change in crystallinity during 48 h of accelerated degradation (n = 3). Data are presented as mean ± SD. *, ** and *** in figures indicate *p* < 0.033, *p* < 0.002 and *p* < 0.001, respectively.

Compression tests were carried out on the degraded samples with their respective controls (Figure 5A–G). Similar to the tests performed on freshly prepared scaffolds, elastic moduli of the porous scaffolds were found to be significantly lower than nonporous scaffolds after the degradation study. Since the degradation and mass loss in the P30 group was faster than that of the P100 group, after 36 h of accelerated degradation, the mechanical properties of P30 scaffolds declined more significantly (Figure 5). The elastic modulus of P30 scaffolds decreased from 1.78 ± 0.26 MPa at 0 h to 0.26 ± 0.06 MPa after 36 h of degradation. P100 scaffolds also showed a reduction in the elastic modulus from 1.33 ± 0.20 MPa at 0 h to 0.39 ± 0.07 MPa after 36 h. These values can be used to guide the tuning of similar scaffolds to the mechanical requirements for a particular tissue regeneration application.

PCL is composed of crystalline (highly ordered) and amorphous (random coil) regions. Crystalline regions are tightly packed by polymer chains and amorphous regions are more susceptible to hydrolysis. Hence, the initial mass loss of the polymer is due to the hydrolytic chain cleavage of the amorphous polymer regions. This leads to an overall increase in the crystallinity of the polymer [77]. The thermal behaviour of the polymer was studied by DSC. The thermal parameters, including the melting temperature (T_m_), melting enthalpy (ΔH_m_) and degree of crystallinity (X_c_), are summarized in Figure 5. The melting point and onset melting point did not show a significant difference before and after the degradation study. After degradation, the scaffolds showed an increase in melting enthalpy, which implies that crystallization was enhanced during degradation (Figure 5M). P30 scaffolds, which showed the fastest degradation in 24 h, showed a significant increase in crystallinity from 47.61 ± 6.44% before degradation to 72.69 ± 1.52% after degradation. This increased crystallinity is expected, in turn, to have implications for the mechanical properties and subsequent degradation rate of the remaining portion of the scaffold.

Figure 5 shows the GPC analysis of PCL scaffolds before and after 36 h of degradation. The increased elution time for the degraded samples indicates that the molecular weight decreased. The M_n_ data showed that the molecular weight of PCL pellets, NP-0Hr, P30-0Hr, P100-0Hr and NP-48Hr were in the same range and measured as 157.07, 163.17, 149.47, 144.17 and 169.61 kDa, respectively. Extra peaks in P30-48Hr and P100-48Hr confirmed the presence of shorter polymer chains due to degradation. P30-48Hr and P100-48Hr M_n_ results showed long molecular chains, with a molecular weight of 154.30 and 148.56 kDa, and shorter molecular chains, with a molecular weight of 2.60 and 2.59 kDa, respectively (Figure S15). P100 scaffolds showed a greater proportion of the longer chains, consistent with the fact that degradation was more advanced for the P30 scaffolds after 48 h.

**Figure 6:**
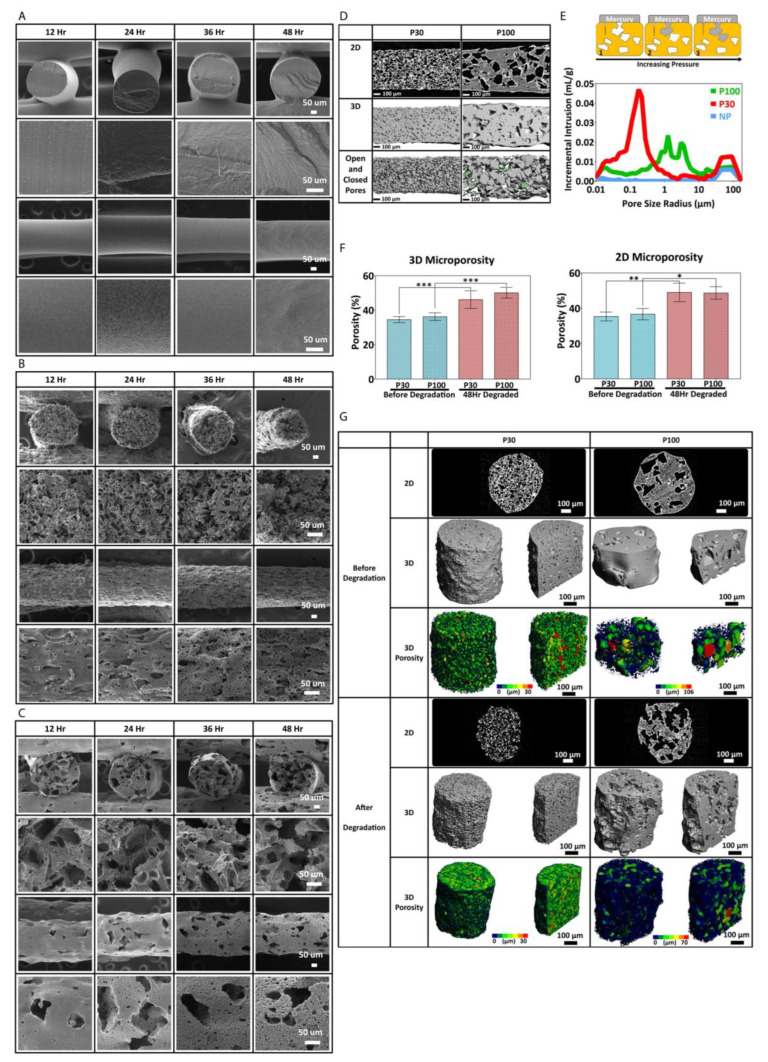
(**A**) Scanning electron microscopy of nonporous scaffolds during degradation. SEM micrographs of P30 (**B**) and P100 (**C**). (**D**) micro-CT and (**E**) mercury intrusion porosimetry (n = 3) show pore interconnectivity of the porous scaffolds (P30 and P100). (**F**) micro-CT 2D and 3D porosimetry before and after degradation (n = 5). (**G**) microporosity 3D visualization and pore size distribution/pore equivalent diameter (µm) of the scaffolds by micro-CT (n = 5). *, ** and *** in figures indicate *p* < 0.033, *p* < 0.002 and *p* < 0.001, respectively.

SEM micrographs show how the surface of the scaffold material and pore structures changed during degradation. P30 scaffolds disintegrated after 48 h of incubation in 2M NaOH, whereas P100 was still structurally stable after 48 h of accelerated degradation (Figure 6B,C). This may be partially due to the increase in brittleness of the P30 scaffolds due to their higher crystallinity after degradation. Hence, it is clear that different levels of microporosity provide different degradation rates as well as different degradation profiles, which would be very useful for tuning and optimizing biodegradable scaffolds to meet a variety of tissue regeneration requirements.

Microporosity increases the fluid flow, scaffold permeability and tortuosity [43,78]. It is known that these parameters directly influence the degradation rate of materials. The interconnectivity of the micropores must also be sufficient to permit cell migration, communication between cells and extracellular matrix (ECM) formation between the pores.

In order to investigate the microporosity, the interconnectivity of the macroporous scaffolds and surface area, micro-CT and mercury intrusion porosimetry were carried out on the scaffolds. Micro-CT and Mercury intrusion porosimetry (MIP) analysis showed that the P30 scaffolds had more interconnected pores in comparison to the P100 scaffolds (Figure 6E). The mercury data show that the P30 scaffolds had several rising peaks at 0.2, 3.6 and 45.1 µm, which indicate the pore size distribution. The rising peaks at 91.6 µm correspond to pore interconnectivity. P100 scaffolds showed rising peaks at 1.3 µm, 3.1 µm, 12.1 µm, and 16.5 µm. NP scaffolds did not show any rising peaks at pore size range smaller than 45.1 µm. These results confirm that higher pore interconnectivity is present in P30 scaffolds in comparison to P100.

Figure 6F shows the 2D porosity obtained from 2D micro-CT slices, analysed by ImageJ software (version 1.53), and the 3D porosity of the scaffolds obtained from 3D micro-CT scans before and after degradation. Both P30 and P100 scaffolds showed a significant increase in 2D and 3D porosity after 48 h of accelerated degradation. Two-dimensional microporosity of P30 and P100 scaffolds increased significantly from 35.33 ± 2.52% to 49 ± 5.29%; and 36.67 ± 3.21% to 48.67 ± 3.51%, respectively. Three-dimensional microporosity of P30 and P100 scaffolds increased significantly from 34.52 ± 1.77% to 46.11 ± 5.12%; and 36.22 ± 2.22% to 50.12 ± 3.12%, respectively. Due to more surface area and interconnectivity facilitating improved hydroxide ion contact, faster degradation is expected for more highly porous scaffolds. In addition, better fluid flow and nutrient exchange in vivo would be enabled by the higher microporosity, which (along with the higher degradation rate) is consistent with the requirements for faster regenerating tissue. Although accelerated degradation experiments allowed for studying the degradation behaviour of the scaffolds over a short period, these studies do not fully capture the complex and dynamic in vivo environment; therefore, in vivo studies are needed to confirm the findings.

It appears from the combined degradation results that scaffolds with smaller, more interconnected micropores would be suited to applications involving relatively rapid tissue repair, such as bone, whereas slower degrading scaffolds with larger but less interconnected micropores may suit slower-regenerating tissues, such as tendon or cartilage. The situation is obviously more complicated than this, and detailed tuning of degradation properties by this method, for a specific application, would require the study of a wide range of discreet porogen sizes. In this study, scaffolds consisting of straight filaments and square pores were investigated to keep the number of experimental groups at a reasonable level. However, it is known that structural tortuosity, which can be altered by changing the size and curvature of the pores of the scaffolds, can influence the permeability and transport properties of scaffolds [80]. This can be used to fine-tune the scaffolds for intended tissue engineering applications.

### 3.4. Cell Culture Study

Rough textures have a significant influence on cell growth and behaviour [81]. Small pores, in the order of a few microns, facilitate cell attachment by providing anchoring sites [82], whereas large pores, in the order of hundreds of microns, facilitate vascularization [13,83,84]. If the pore size is significantly larger than the cell size, cell attachment will decrease due to the large surface discontinuities. Otherwise, surface roughness usually increases cell attachment [85,86]. Moreover, a porous surface leads to a better mechanical interlocking between the scaffold and host tissue [87]. Porous structures lead to a more moderate immune reaction and thinner fibrous capsule formation [79].

**Figure 7:**
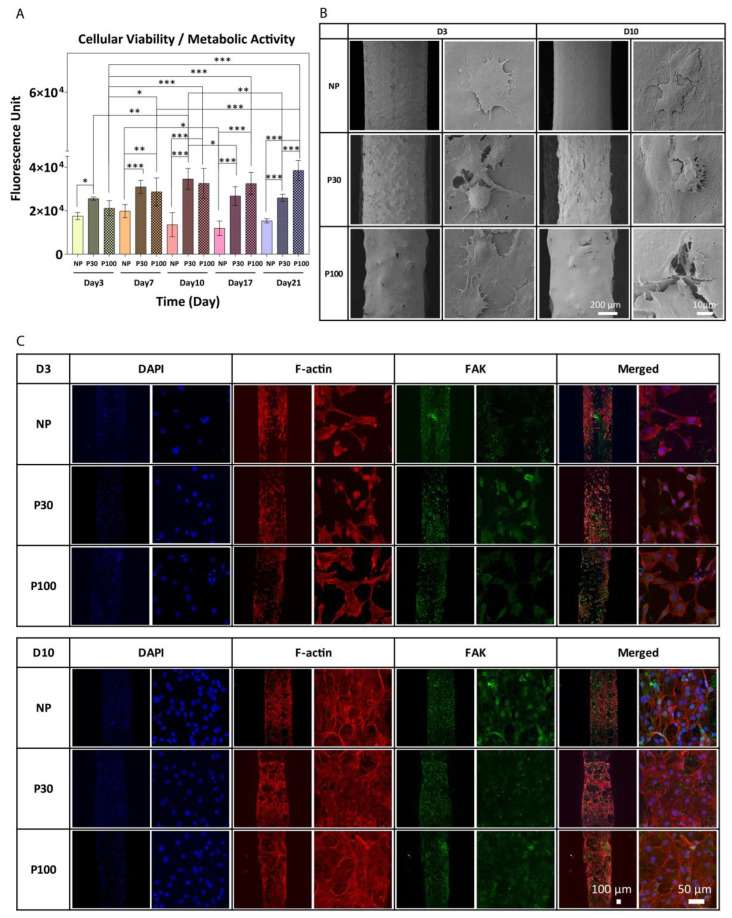
(**A**) PrestoBlue cell metabolic activity. Data showed an increase in cell metabolic activity from Day 3 to Day 21; data are presented as mean ± SD. *, ** and *** indicate *p* < 0.033, *p* < 0.002 and *p* < 0.001 respectively. (**B**) SEM images of cultured fibroblast cells after 3 and 10 days of culture. (**C**) Representative confocal microscopy images of fibroblast cells cultured on NP, P30 and P100 scaffolds at Day 3 and Day 10. Cells were labelled with antibodies to visualize Nuclei (DAPI; blue), FAK (green) and F-actin (Phalloidin; red).

The PrestoBlue data show a significant increase in cell metabolic activity in cells seeded on P30 and P100 scaffolds, from Day 3 to Day 21 (Figure 7A). Figure 7B shows representative SEM images of attached cells on the scaffolds at Day 3 and Day 10 after seeding. On all three types of surfaces with different topography and surface roughness, F-actin and FAK expression were detected and confirmed by immunofluorescent staining and confocal microscopy (Figure 7C). Expression of FAK and F-actin confirms fibroblast cell spreading and migration. The data confirmed the suitability of the scaffold surface for cell attachment and proliferation. While the study demonstrates improved cell attachment, proliferation, and extracellular matrix production, these tests were only performed in vitro; therefore, further studies are necessary to confirm the efficacy of these scaffolds in vivo.

### 3.5. Blood Clotting Study

**Figure 8:**
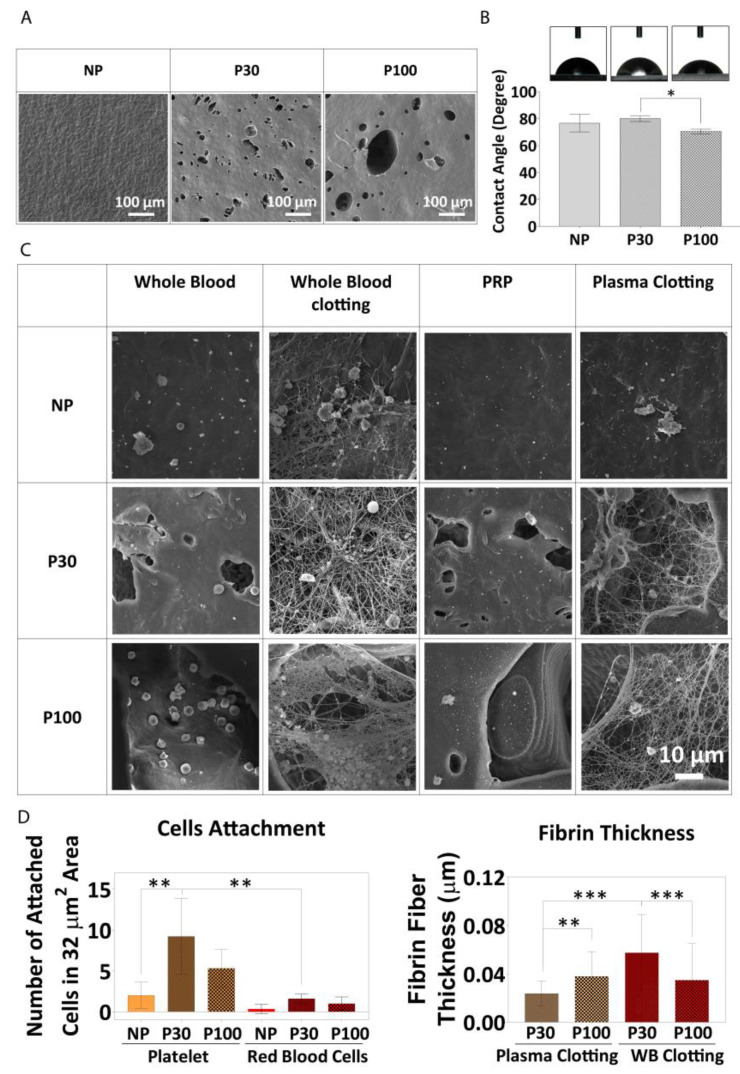
(**A**) SEM of PCL films showing the surface topography before the experiment. (**B**) Water contact angle (wettability) of PCL films (n = 4). (**C**) Whole blood attachment and whole blood clotting on PCL films. (**D**) Fibre diameter, measured from SEM micrographs, (n = 6). Data are presented as mean ± SD. *, ** and *** in figures indicate *p* < 0.033, *p* < 0.002 and *p* < 0.001, respectively.

The implantation of a biomaterial into the body induces injury and blood–material interactions. Within seconds, proteins of the blood plasma with high affinity to surfaces adsorb to the biomaterial to form a very sparse provisional protein matrix of 2–5 nm. This matrix finally develops into a fibrin-dominated thrombus with region-specific differences. Since all involved cells interact with the provisional matrix rather than the foreign body surface itself, the matrix composition is assumed to be of major relevance for all subsequent in vivo events [88]. The composition of the early provisional matrix and the final thrombus depends on several features, including the physicochemical properties of the biomaterial surface and the blood plasma composition. Depending on the type of implant, plasma-rich platelet (PRP) [89] attachment on the surface of the implant and blood clotting can be considered an advantage or a disadvantage. Blood clotting and platelet activation induce the release of cytokines and chemokines [90]. Clotted blood has vital elements to modulate the initial immune response [91]. PRP has bioactive properties as it contains fibroblast growth factors and platelet-derived growth factors [92], which enhance cell adhesion and vascularization and stimulate tissue regeneration [93,94]. Thus, blood–polymer interactions, blood clotting, and platelet adhesion are important factors in scaffold characterization [95]. Therefore, the interaction between PCL and blood (whole blood attachment, PRP attachment, whole blood clotting, and plasma clotting) was investigated (Figure 8).

Blood–polymer interaction might be affected by surface topography, surface charge, and wettability. The surface hydrophilicity controls the response of cells to the surface and the interaction with proteins. Figure 8B shows surface wettability in the different groups. All three scaffold groups showed contact angles less than 90 degrees, indicating reasonably good wettability and a potentially cell-friendly surface, and this was borne out by the cell culture study described above. NP, P30, and P100 groups showed contact angles of 76.64 ± 6.55°, 80.00 ± 2.16° and 70.35 ± 1.76°, respectively.

It has been shown that surface chemical functionalization improves blood clotting and PRP attachment on implant surfaces [88,96]. However, there is a research gap to investigate the influence of topography on blood clotting in the vicinity of scaffolds with microporous architectures. The microstructure of the pores provided a suitable space for entrapping and anchoring blood cells (Figure 8A). The number of red blood cells (RBCs) attached to the NP group (0.33 ± 0.57 cells within 32 µm^2^ area) was lower than the number of cells attached to the P30 group (1.6 ± 0.55 cells within 32 µm^2^ area) and P100 group (1.0 ± 0.82 cells within 32 µm^2^ area). No significant difference was found between P30 and P100 groups in terms of the number of RBCs attached (Figure 8C). The number of platelets from PRP attached to the surface of the NP group (2 ± 1.63 cells within 32 µm^2^ area) was less than the number attached to the surface of P30 group (9.25 ± 4.65 cells within 32 µm^2^ area) and P100 group (5.33 ± 2.31 cells within 32 µm^2^ area), supporting the hypothesis that porous scaffolds show a better response to platelet attachment, and would, in turn, be expected to show more growth factor release in an in vivo setting. Whole blood clotting and plasma clotting led to thicker fibrin fibres on P30 (0.06 ± 0.03 and 0.02 ± 0.01 µm, respectively) and P100 scaffolds (0.03 ± 0.03 and 0.04 ± 0.02 µm, respectively) than on NP scaffolds (Figure 8D). Between the P30 and P100 groups, P30 showed lower thickness in plasma clot (0.02 ± 0.01 µm) (loose clot) and higher thickness in whole blood clot (firm clot) (0.06 ± 0.03 µm). The data did not show any significant difference between P100 plasma clot fibrin fibre thickness (0.04 ± 0.02 µm) and P100 whole blood clot fibrin fibre thickness (0.03 ± 0.03 µm), which confirms the same firmness of the clot in P100 groups (Figure 8D).

### 3.6. In Vitro Drug Release from Multiphasic Scaffolds

The local release profile of an antibiotic from a tissue implant should show a high initial release rate, in order to prevent the initial bacterial contamination, followed by a sustained release to inhibit the occurrence of latent infection [97]. If the drug load is released too quickly, the antibiotic loading is exhausted before the infection is stopped. The high surface area of microporous scaffold designs makes them potentially good carriers for controlled drug release due to high drug loading and mass transport capacities [67,98]. The controllable pore size and pore distribution in multiphasic scaffolds can provide an extra level of tunability necessary for drug delivery applications. In this study, the drug release profiles of NP, P30 and P100 scaffolds were investigated. Cefazolin sodium, a highly water-soluble antibiotic, was used as the model drug in this study. It is clinically effective against infections caused by Gram-negative and Gram-positive bacteria. Based on a study by Visscher et al., cefazolin concentrations of up to 100 µg/mL did not show any cytotoxic effect in vitro on 3T3 fibroblast cells and neither on in vitro blood clot formation on the scaffolds. Hence, the concentrations of cefazolin used in this study are considered safe [99].

**Figure 9:**
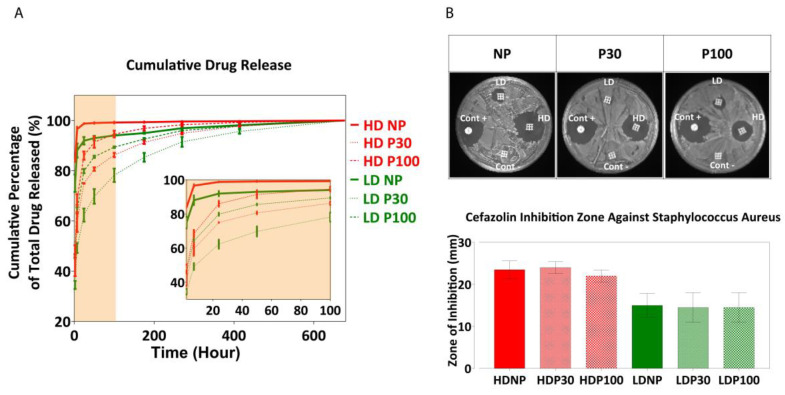
(**A**) Drug release profile. Data are presented as mean ± SD (n = 8). (**B**) Antibacterial inhibition zone. Positive control (Cont+) is Oxoid^TM^ cefazolin antimicrobial susceptibility disks (30 µg); Negative control (Cont−) is drug-free scaffolds. Data are presented as mean ± SD (n = 3).

Scaffolds exhibited significantly reduced burst release of cefazolin when compared to non-porous scaffolds and maintained a sustained release of the drug for a prolonged period of up to 600 h (Figure 9A). The loading efficiency was calculated by subtracting the drug loss in the loading solution from the total drug loaded onto the scaffolds. Among the high-dose groups (0.6 mg/mL) and low-dose (0.06 mg/mL) groups, NP, P30, and P100 did not show any significant differences in loading efficiency.

Figure S24D,E shows cumulative drug release compared between different types of scaffolds at different timepoints of the release (2 h, 7 h, 24 h and 50 h). At the beginning, there was a significant difference between the amounts of drug released from NP, P30 and P100 scaffolds, when loaded with both low- and high-dose concentrations. From Day 11 (270 h), no significant difference was observed in the amount of drug released from different scaffolds.

The antibacterial efficacy of the drug-loaded scaffolds was tested against *S. aureus* by the zone of inhibition on agar plates. *S. aureus* is a Gram-positive bacteria and one of the most common pathogens found in surgical sites and the most common cause of surgical site infection in patients undergoing hip and knee arthroplasty [100]. The presence of such infections would also increase the chance of bacteremia [101]. For these reasons, *S. aureus* was the focus of this part of the research. The zone of inhibition was measured after 24 h (Figure 9B). A dose-dependent zone of inhibition was observed, indicating the ability to successfully modulate the antibacterial activity using this scaffold-loaded approach to drug delivery.

## 4. Conclusions

The combined AM/SCPL-based manufacturing technique used in this study facilitated the development of scaffolds with multiscale morphology that are potentially suited to dental tissue engineering. The use of 3D printing technology allowed for manufacturing scaffold architectures consisting of well-defined macroscale structural features and it can facilitate the development of patient- and tissue-specific scaffold designs in the future. In parallel, the incorporation of the porogen-leaching technique allowed for tuning the surface properties and micro-scale pore structure of the scaffolds. The comprehensive materiome characterisation showed that these scaffolds were highly tuneable in terms of not only their design but also their mechanical properties and porosity, as well as degradation and drug release kinetics. Owing to the synergy created by these two manufacturing approaches, this level of control was achieved without compromising the reproducibility and speed in manufacturing as well as the achievable scaffold size, which are important aspects that enable broadening the application range as well as strengthening the clinical translation potential of such TE scaffolds. Favourable cell and blood reactions as well as a sustained drug release profile following a mild burst release observed for these scaffolds guarantee them as future candidates for local drug delivery applications. Future studies will evaluate the in vivo performance of these scaffolds for dental tissue regeneration applications.

## Data Availability

Data is contained within the article or Supplementary Material.

## Supplementary Materials

The following supporting information can be downloaded at: https://www.mdpi.com/article/10.3390/pharmaceutics15051340/s1, Figure S1. Macroscopic image of the printed scaffold; Figure S2. Schematic presentation of the Bioextruder 3D printer; Figure S3. Schematic representation of manufacturing porous scaffolds; Figure S4. Thermogravimetric analysis of polymer-salt composite scaffolds for prediction of Bioextruder 3D printing success (n = 3); Figure S5. Effect of the printer needle size on mechanical properties; Figure S6. Porogen size measurement and analysis; Figure S7. X-ray diffraction spectra; Figure S8. Energy Dispersive X-ray spectroscopy mapping does not show peaks corresponding to PBS (Na, P and K) in leached samples; Figure S9. SEM micrographs of leached NP scaffolds show that the NaOH 0.01M did not have any effects on the scaffolds after 16 days; Figure S10. MicroCT evaluation of P30 and P100 scaffolds by Aviso software (version 9.5.0); Figure S11. Microscopic analysis of the leaching process; Figure S12. (A) Leaching Scaffolds in 0.01M NaOH. (B) ICP-OES analysis of the scaffolds confirmed complete salt leaching (n = 3); Figure S13. Chloroform tracing inside the printed scaffolds by NMR; Figure S14. Scaffold accelerated degradation analysis; Figure S15. (A) NP scaffold degradation after 48 h of incubation in 2M NaOH. (B) microCT of NP 48-h degraded samples did not show any signs of degradation inside each filament; Figure S16. Gel permeation chromatography (GPC) analysis shows shorter polymer chains in 48-h degraded P30 and P100 scaffolds; Figure S17. Fibroblast cell response analysis; Figure S18. Film surface characterization; Figure S19. Blood cell interaction with PCL films; Figure S20. Whole blood clotting on PCL films; Figure S21. Plasma clotting on PCL films; Figure S22. PRP attachment to PCL films; Figure S23. Cefazolin drug release; Figure S24. Drug release kinetics; Figure S25. Cefazolin tracing inside the dug released scaffolds by ^1^H-NMR. References [102,103,104,105] have been cited in Supplementary Materials.
