# Supplementary material for: Microporous/Macroporous Polycaprolactone Scaffolds for Dental Applications
Source: Pharmaceutics. 2023 Apr 26;15(5):1340. doi: 10.3390/pharmaceutics15051340 (PMC10220766; doi:10.3390/pharmaceutics15051340)
Supplement: Supplementary file 1 [file pharmaceutics-15-01340-s001.zip › pharmaceutics-2224076-supplementary.pdf]

Microporous/macroporous polycaprolactone scaffolds for dental applications

*Tara Shabab, Onur Bas, Bronwin L. Dargaville, Akhilandeshwari Ravichandran, Phong A. Tran, Dietmar W. Hutmacher**

Supplementary Information

1. Supplementary Figures

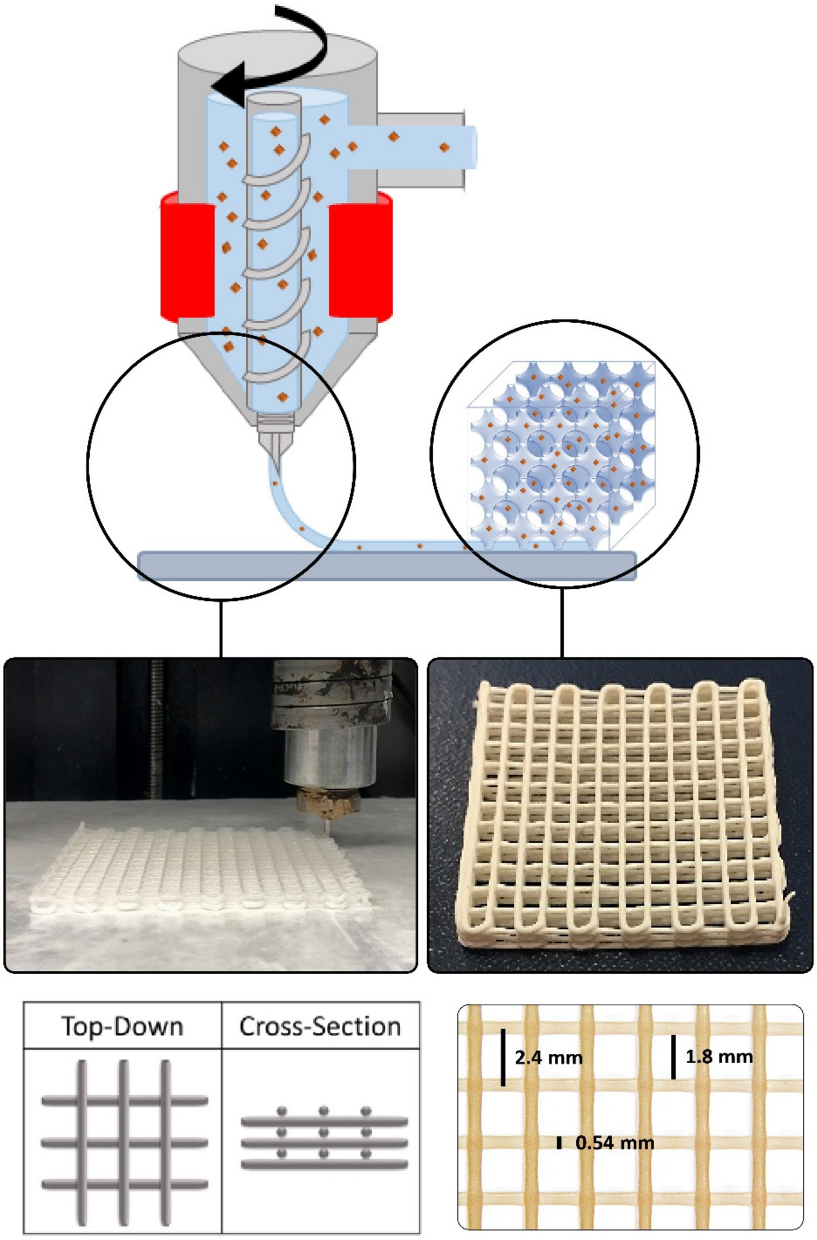

Groups	NP	P30	P100
Porogen Size	_	<30 µm	90 < < 150

Figure S1. Macroscopic image of the printed scaffold.

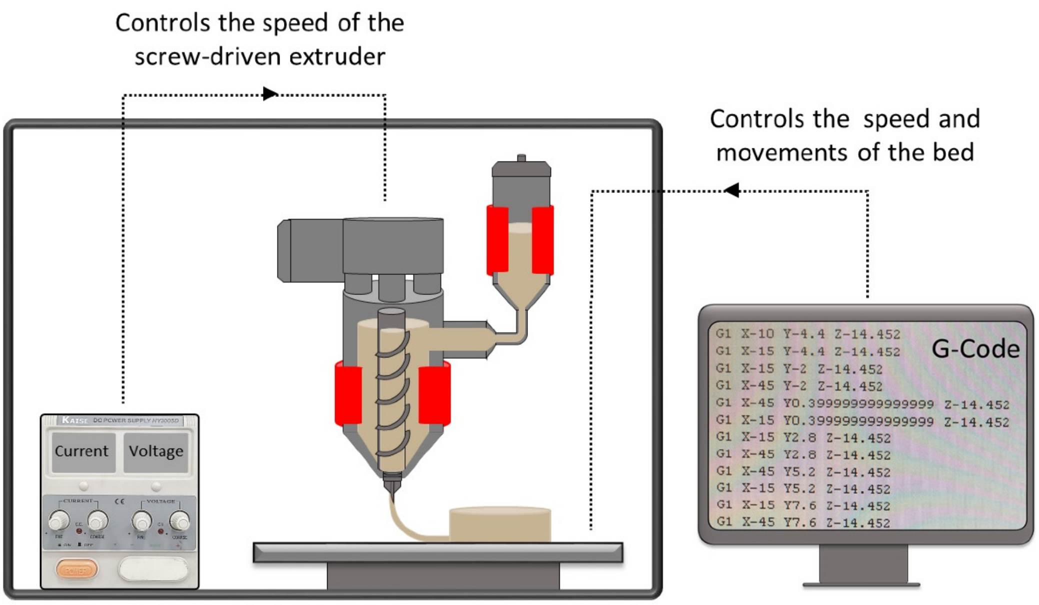

Needle Size	G19 (Inner Diameter: 0.7 mm)
Strut Thickness<br>Filament Distance	0.54 mm (540 µm)<br>2.4 mm (2400 µm)
Number of Layers	6
Strut Angles/Laydown Pattern	0°/90°
Printing Speed<br>Macroporosity	6.5 mm/sec<br>80% - Quadrangular Pores
Microporosity	45% - Polygonal Pores

Figure S2. Schematic presentation of the Bioextruder 3D printer. In the Bioextruder, the voltage and the current control the speed of the screw-driven extruder. The pneumatic pressure helps the dispensing of the thermoplastic material too. The bed movement and the print speed can be controlled by the software [1]. In order to prevent the scaffold from slumping, printing speed, macroporosity and the needle size should be optimized. Parameters were set to achieve 80-85 % macroporosity which is also confirmed by microCT analysis. Needle G19 was the optimized needle in this study. Printing with a bigger needle (G17) was not successful due to lower pressure.

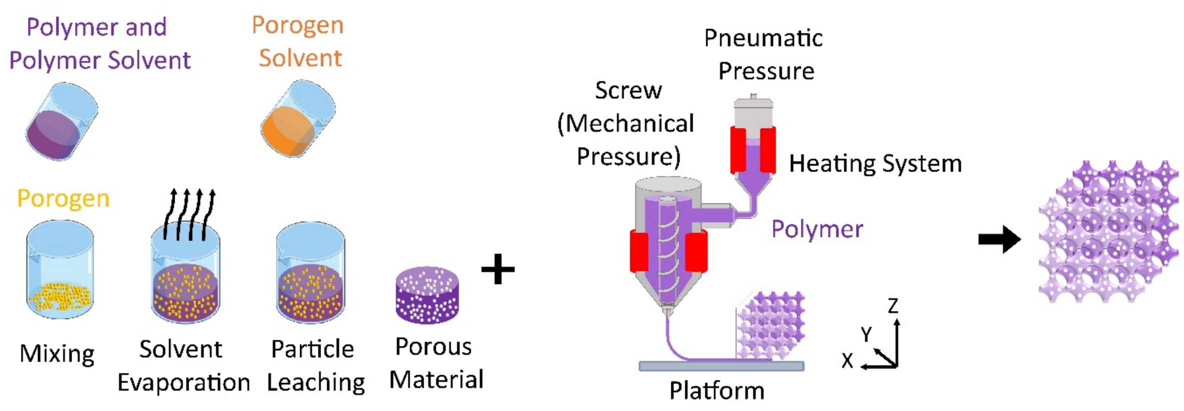

	Solvent Casting Particle Leaching	Melt Extrusion-Based 3D Printing
Pros	• Low cost<br>• Highly Porous Microstructure<br>• High Pore Interconnectivity	• CAD Anatomically-Shaped Scaffolds<br>• High Control on Micro and Macro 3D Architecture<br>• High Control on Macroporosity<br>• Mixed Materials Printing<br>• Reproducibility<br>• High Speed of Fabrication
Cons	• Requires Solvent<br>• Poor Mechanical Strength<br>• Casting Thick 3D Material is not Possible Due to Difficulty in Salt Removal<br>• The Exact Size and Shapes of the Particles are not Controllable	• No Control in Microstructures<br>• High Operating Temperature

3D-Printed Hierarchical Highly Porous Architecture Which is Reproducible and Multifunctional

Figure S3. Schematic representation of manufacturing porous scaffolds. The chart shows the advantages and the problems of each technique [2-6]. With the salt removed, the resulting 3D-polymer has its initial architecture but entirely hierarchical porous [7].

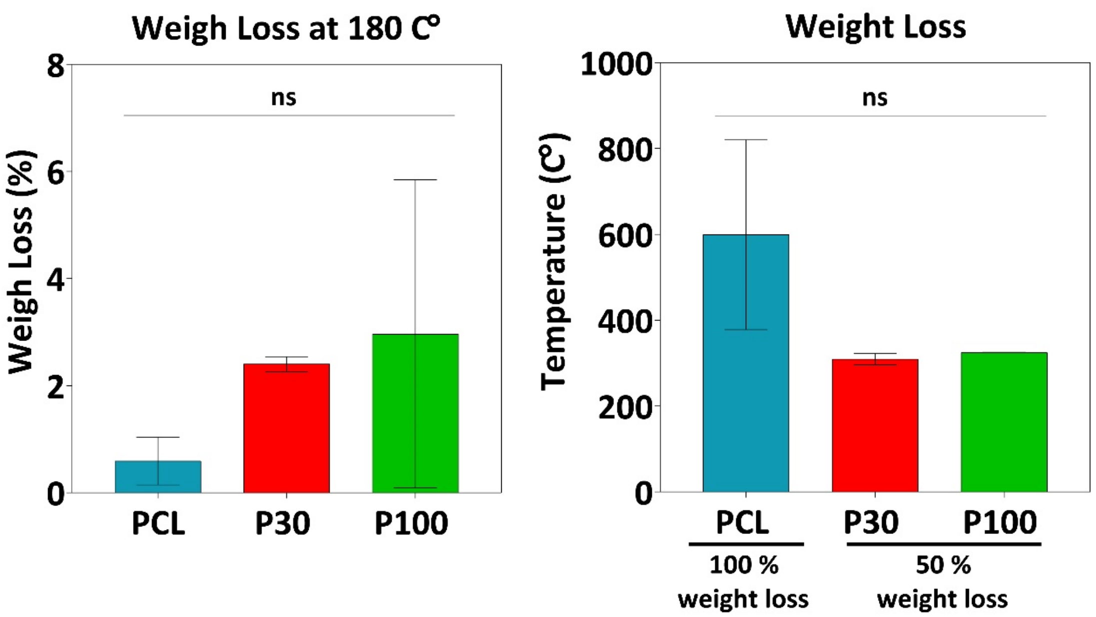

Figure S4. Thermogravimetric analysis of polymer-salt composite scaffolds for prediction of Bioextruder 3D printing success (n=3).

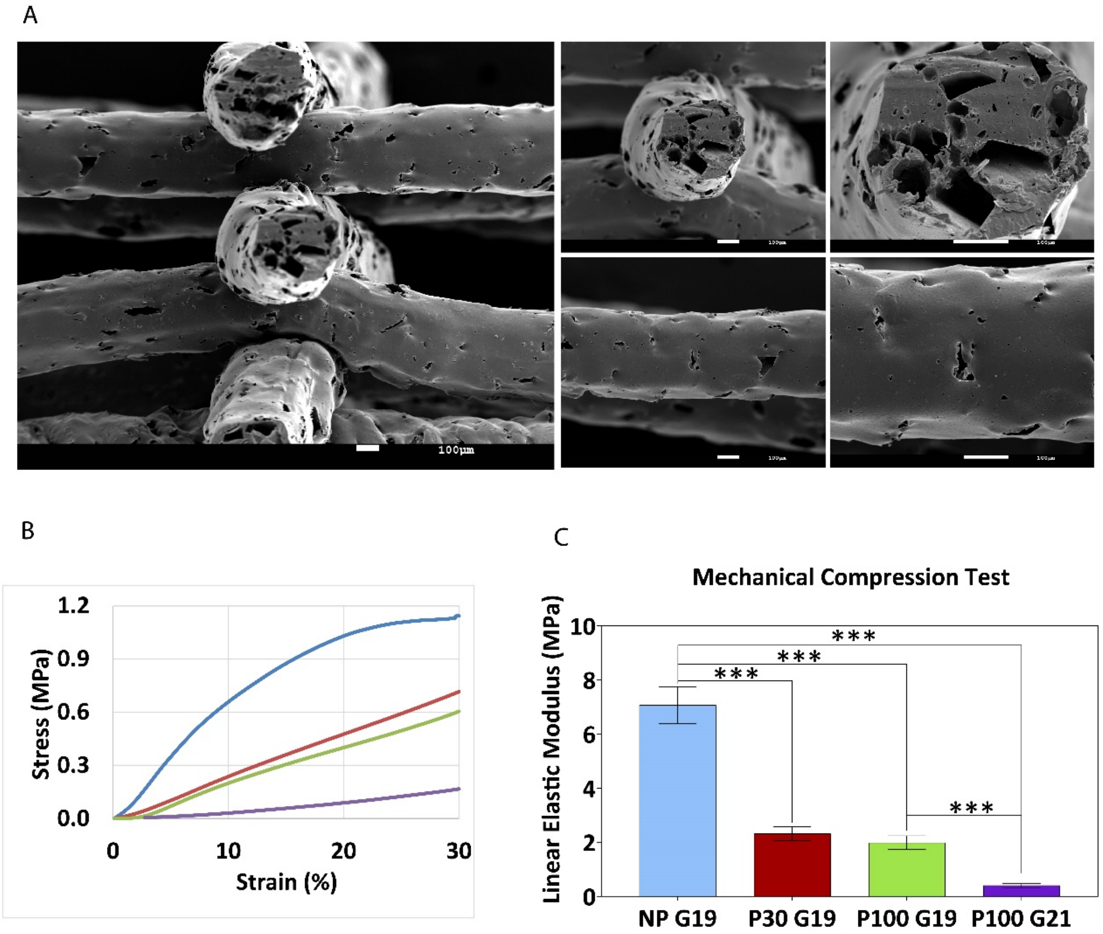

Figure S5. Effect of the printer needle size in mechanical properties. Printing with a smaller needle size (G21) resulted in slumping due to heavy porogen and small strut size. Furthermore, the scaffold did not show suitable mechanical properties (B-C). *** in figures indicate $p < 0.001$, respectively.

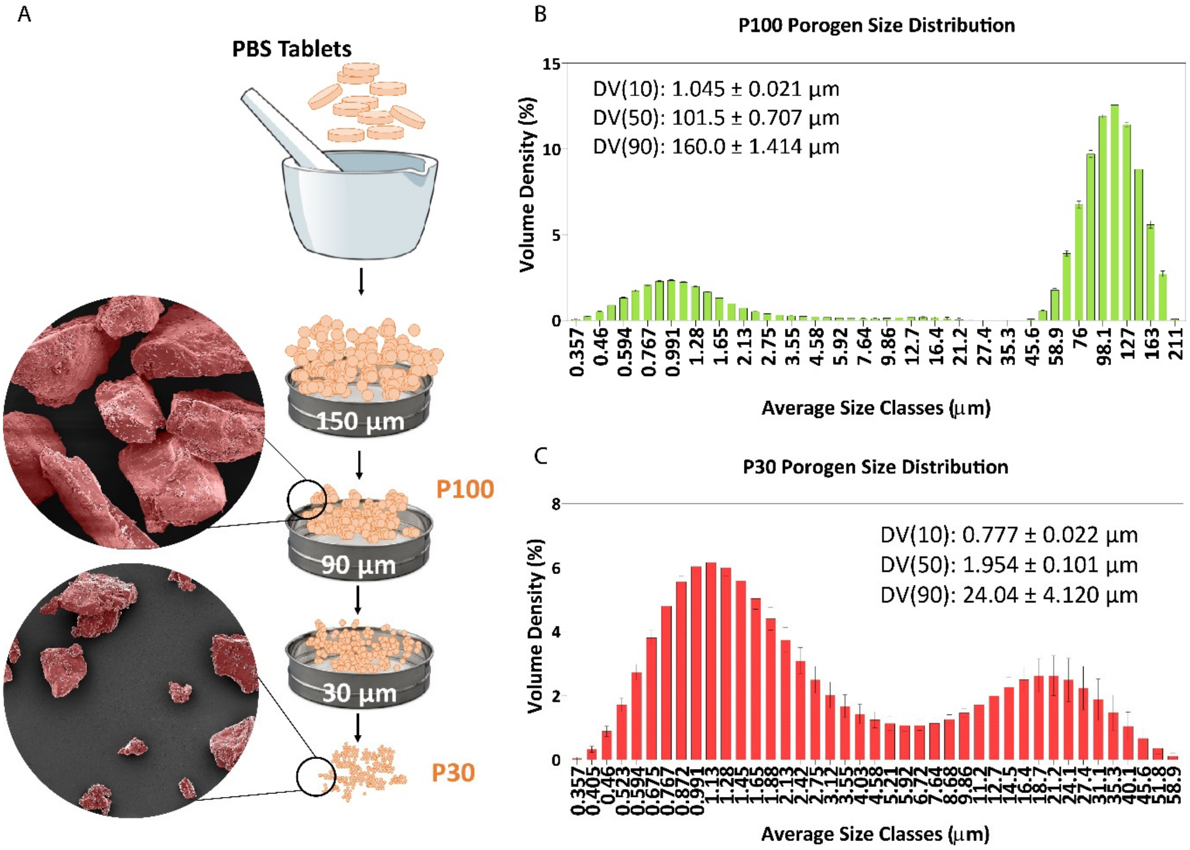

Figure S6. Porogen size measurement and analysis. (A) schematic presentation of porogen preparation and SEM analysis. (B) porogen size measurement, P30 (B) and P100 (C) by dynamic light scattering.

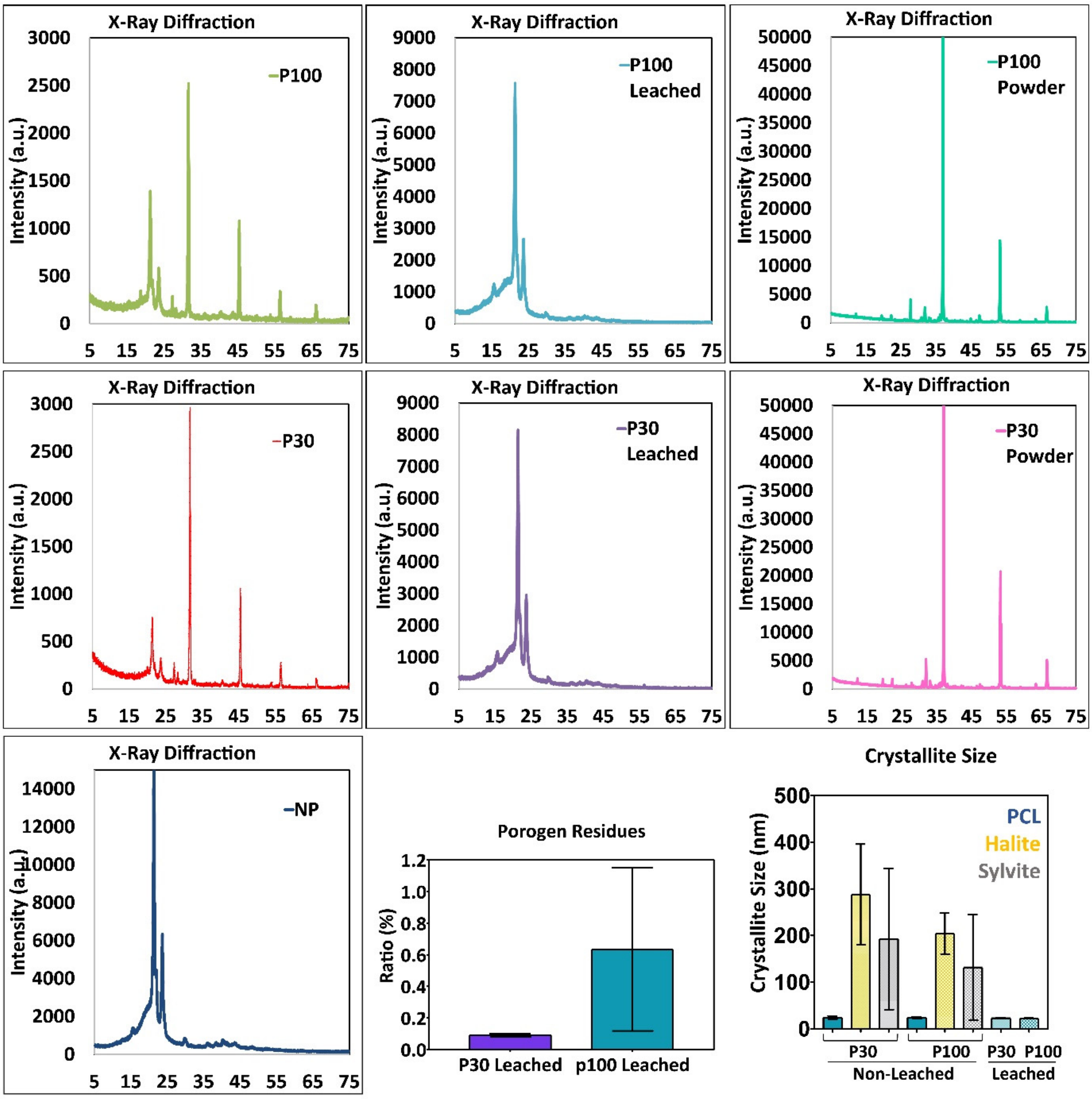

Figure S7. X-ray diffraction spectra. The figure shows X-ray diffraction spectra, crystallite sizes, and porogen residues on leached samples.

**P30 Cross Section**	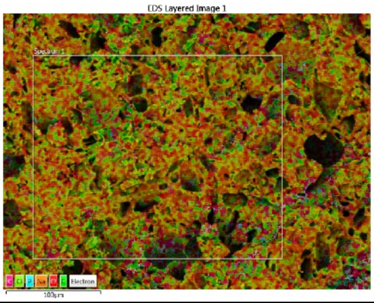	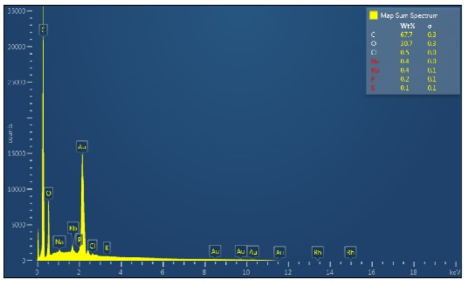
**P100 Cross Section**	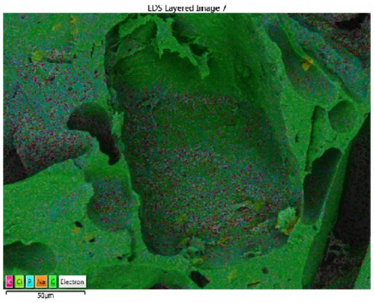	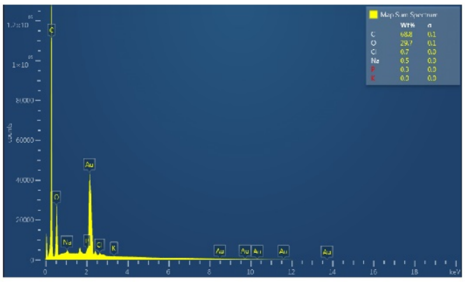

Figure S8. Energy Dispersive X-ray spectroscopy mapping does not show peaks corresponding to PBS (Na, P and K) in leached samples.

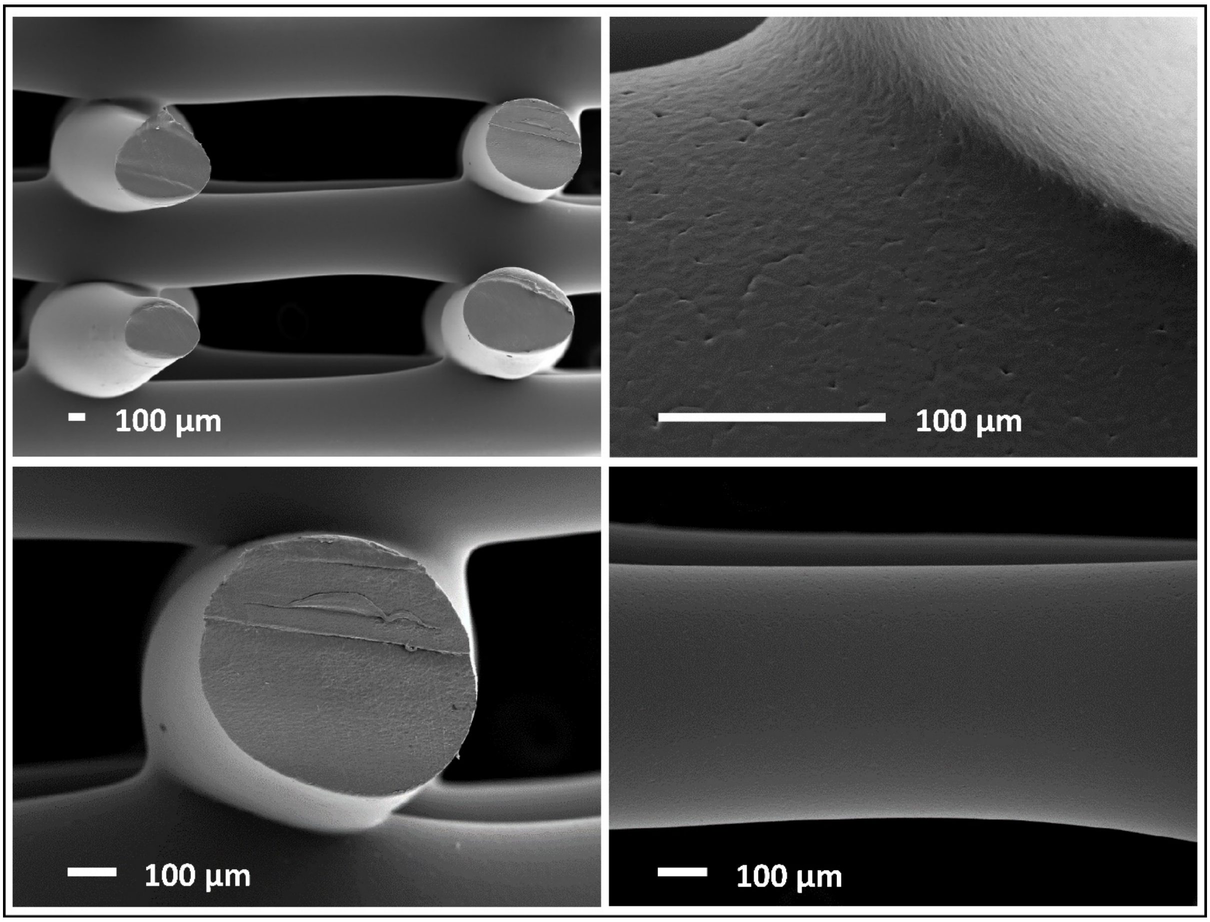

Figure S9. SEM micrographs of leached NP scaffolds show that the NaOH 0.01M did not have any effects on the scaffolds after 16 days.

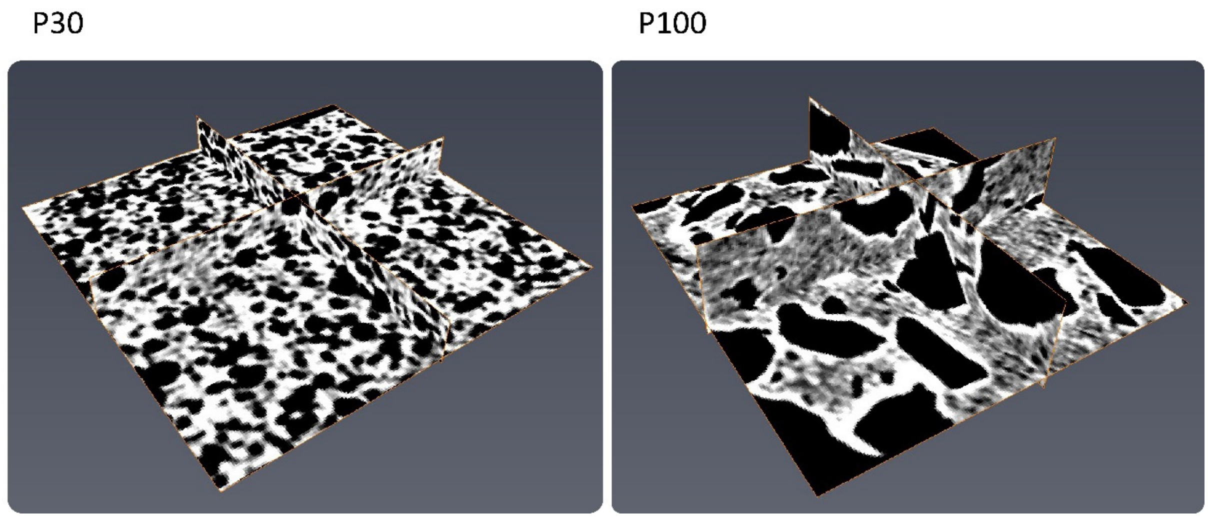

Figure S10. MicroCT evaluation of P30 and P100 scaffolds by Aviso software (version 9.5.0).

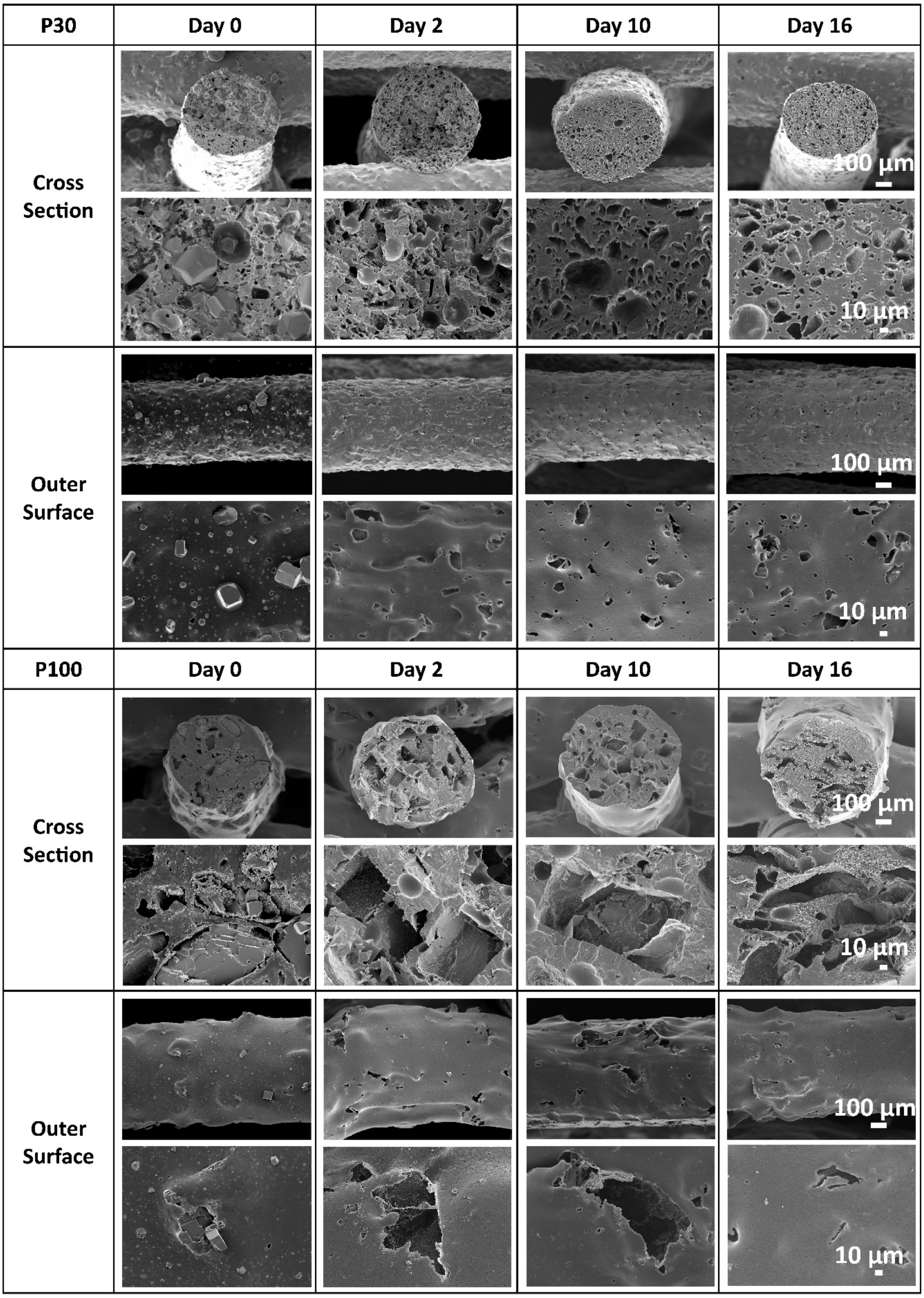

Figure S11. Microscopic analysis of the leaching process. SEM Micrographs show the leaching process for 16 days in P30 and P100 scaffolds.

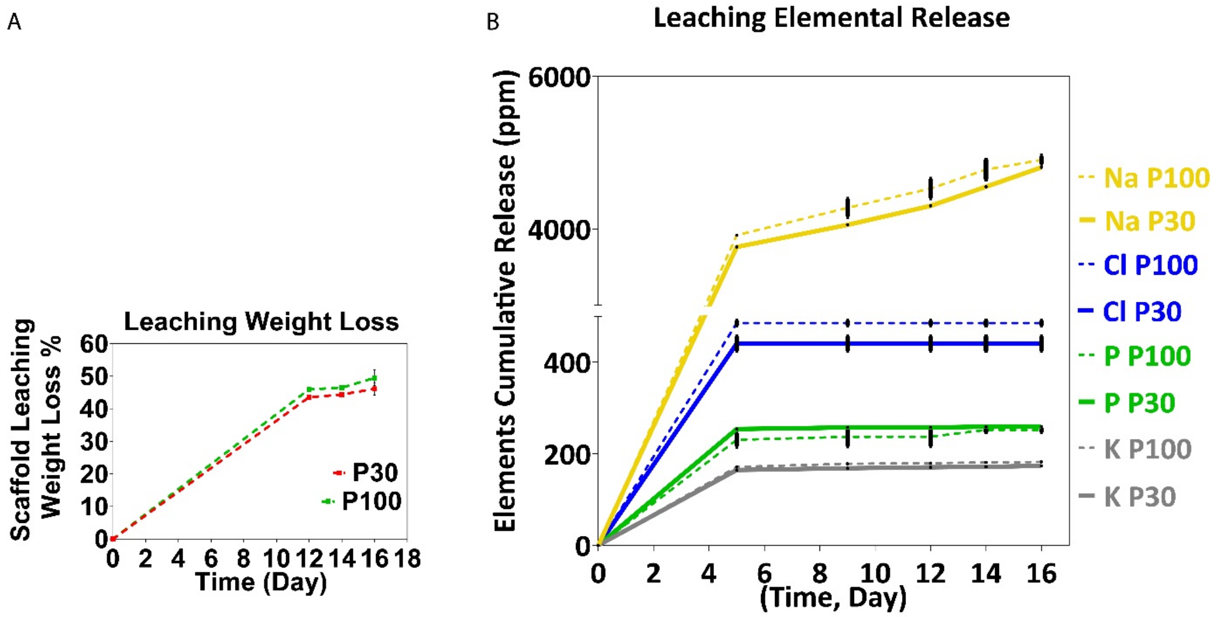

Figure S12. (A) Leaching Scaffolds in 0.01M NaOH. 45% weight loss confirmed PBS leaching (n=4). (B) ICP-OES analysis of the scaffolds confirmed complete salt leaching (n=3).

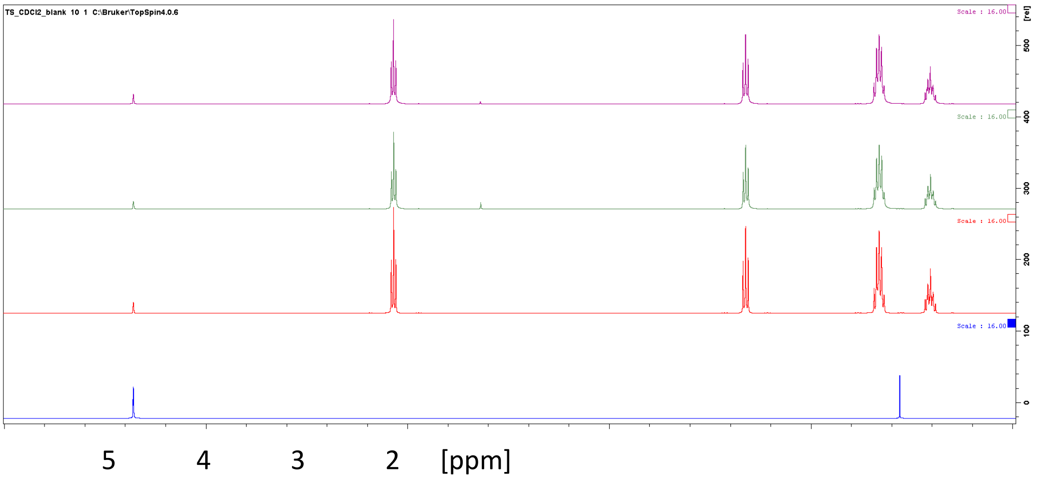

Figure S13. Chloroform tracing inside the printed scaffolds by NMR. The ^{1}H-NMR showed no traces or picks of chloroform ($CHCl_3$) inside the scaffolds which confirms complete chloroform evaporation during the film process.

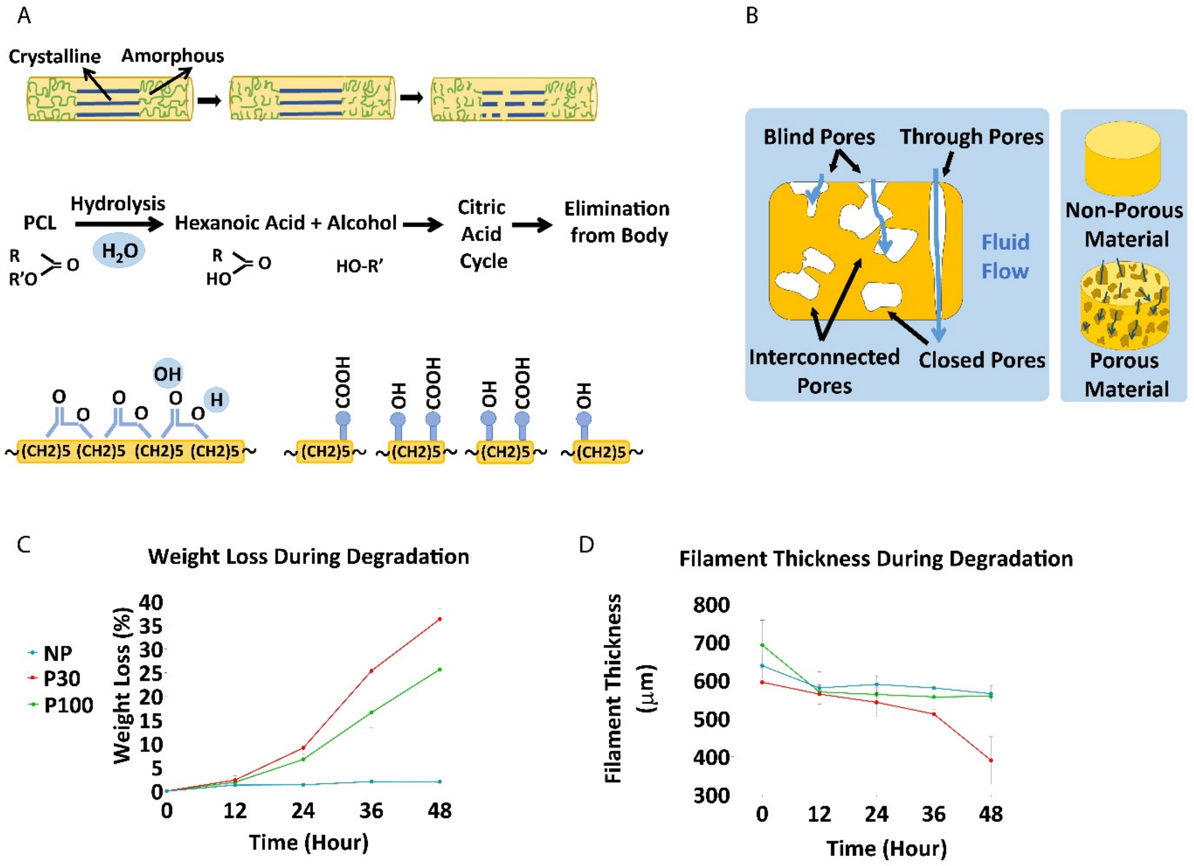

Figure S14. Scaffold accelerated degradation analysis. (A) Schematic illustration of PCL hydrolysis and degradation process. The schematic shows the degradation of the amorphous and crystalline regions of the polymer [8, 9]. (B) Schematic illustration showing the fluid flow in porous scaffolds and its effects on the degradation. (C) The figures show the scaffold weight loss (n=5) and the reduction in the diameter of the filaments (D) during the degradation process (n=5).

A

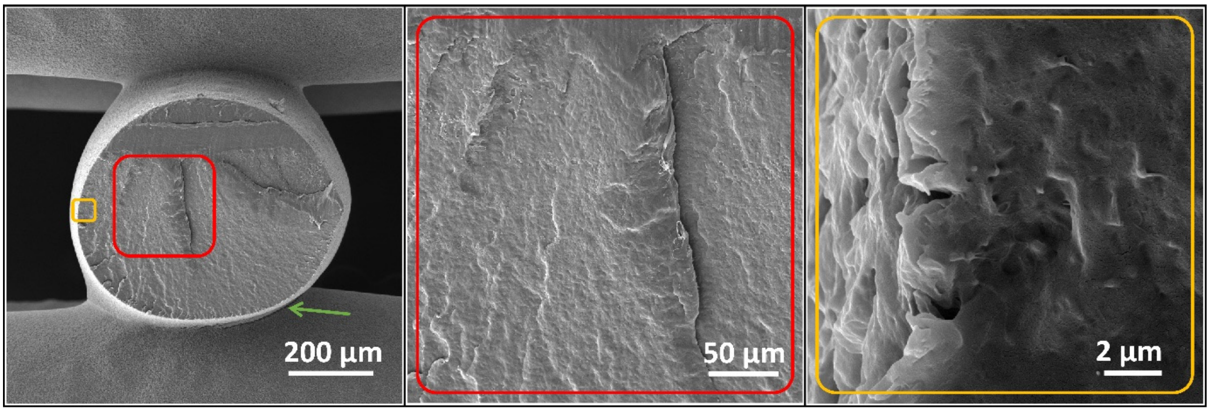

B

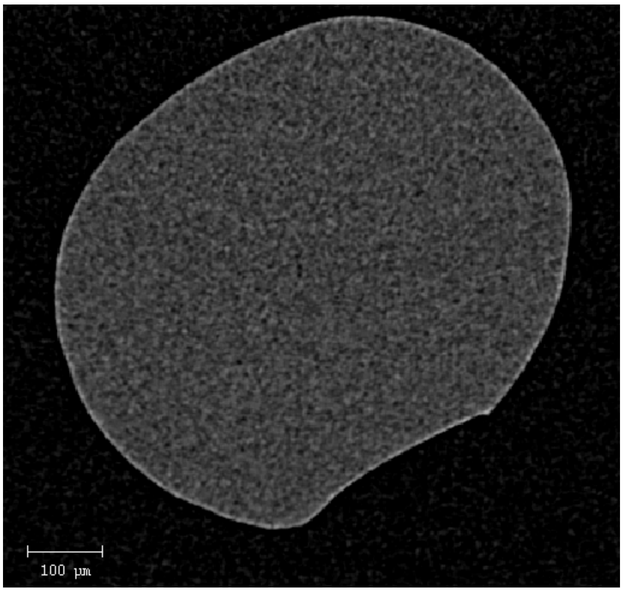

Figure S15. (A) NP scaffold degradation after 48 hours of incubation in 2M NaOH. The green arrow shows the crack on the filament junction. The red rectangle shows the cross-section of the scaffold. The yellow rectangle shows the edge of the cross-section of the filament. The NaOH has not penetrated into the entire filament completely. Most of the degradation is just on the surface and surface erosion can be identified. (B) microCT of NP 48-hour degraded samples did not show any signs of degradation inside each filament.

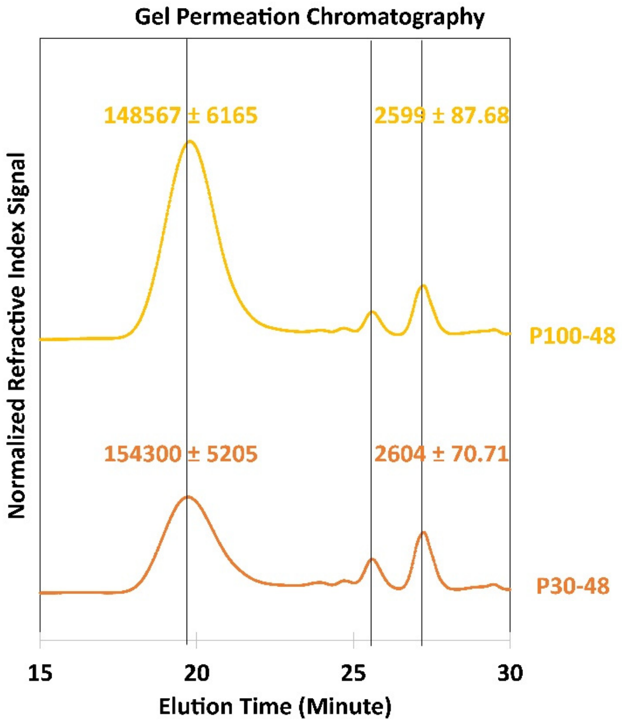

Figure S16. Gel permeation chromatography GPC analysis shows shorter polymer chains in 48-hour degraded P30 and P100 scaffolds.

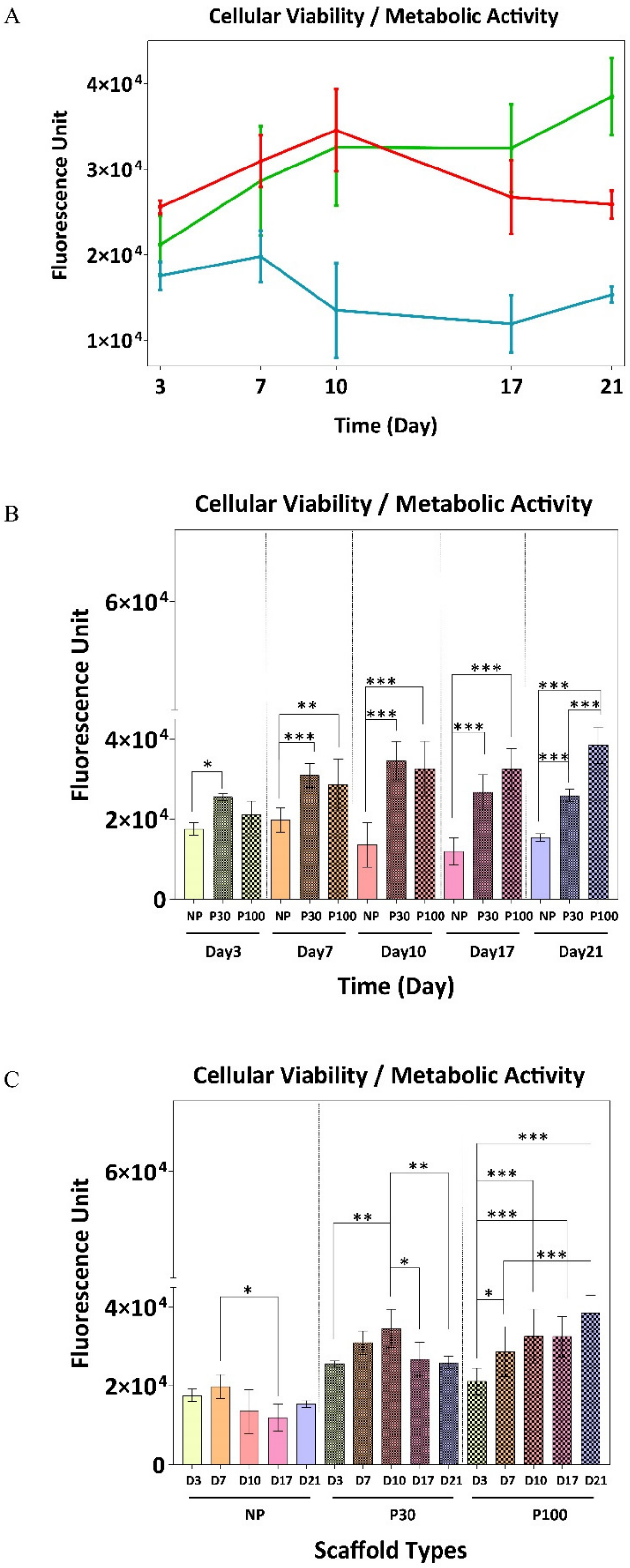

Figure S17. Fibroblast cell response analysis. (A) PrestoBlue analysis based on culture time. (B) Fibroblast cell experiment *in vitro* analysis based on culture time. (C) Fibroblast cell experiment *in vitro* analysis based on the types of scaffolds. *, ** and *** in figures indicate $p < 0.033$, $p < 0.002$ and $p < 0.001$, respectively.

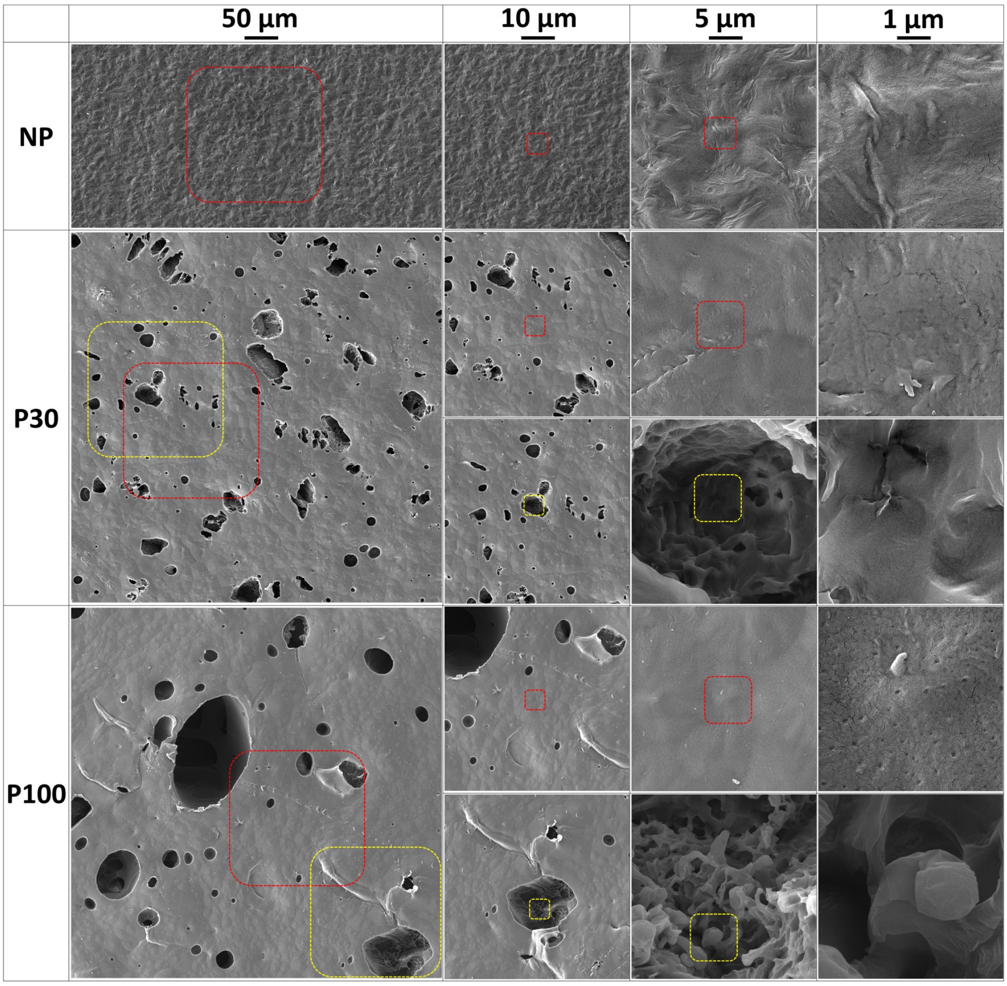

Figure S18. Film surface characterization. SEM of PCL films shows the surface topography before the experiment. The red squares and yellow squares show the surface and inside the pores structures, respectively.

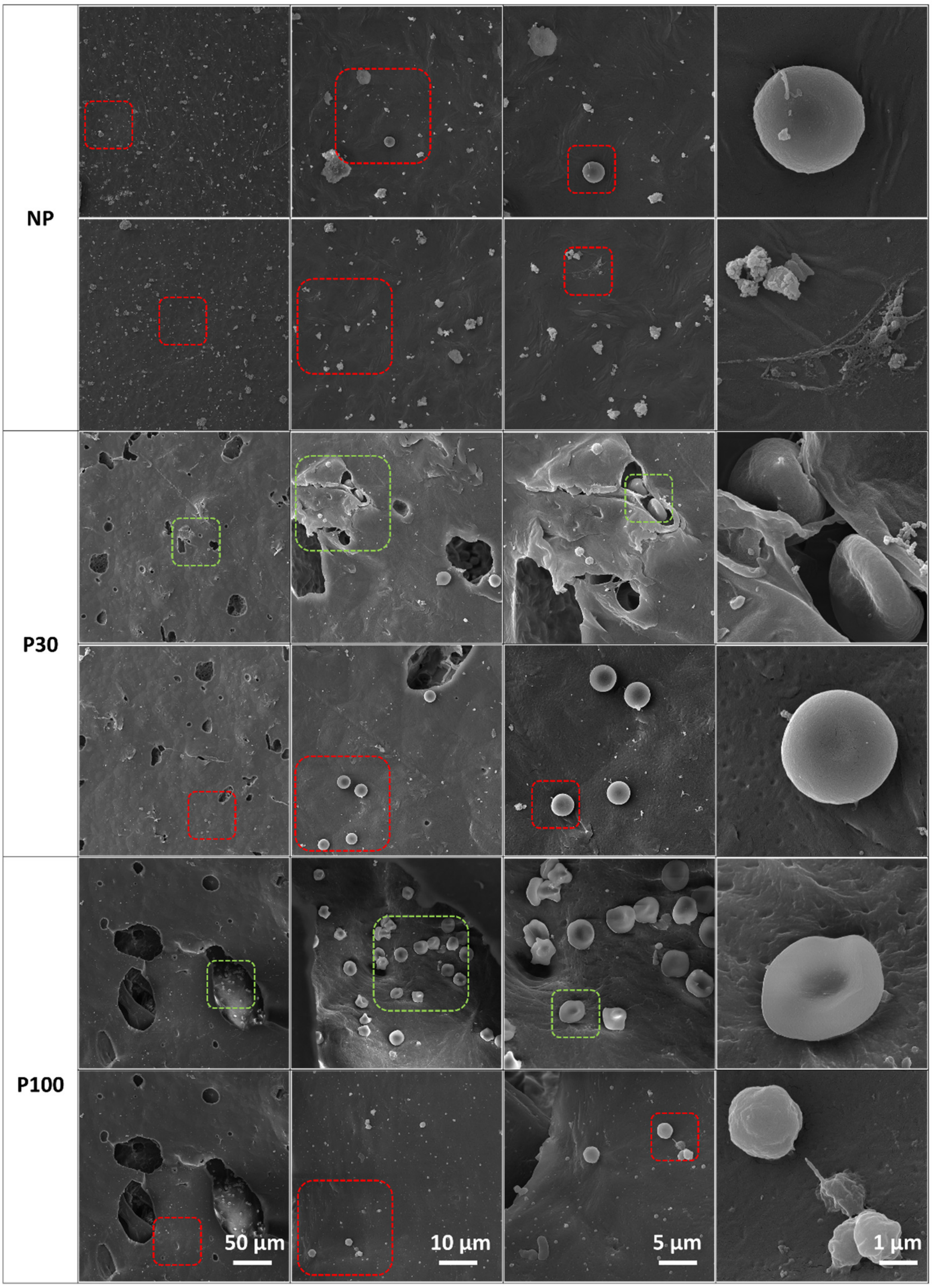

Figure S19. Blood cell interaction with PCL films. The microscopic analysis shows the blood cells attachment on the surface and inside the pores on NP, P30 and P100 films. The red squares show the magnified area. The pore structures provide anchoring sites to entrap blood cells.

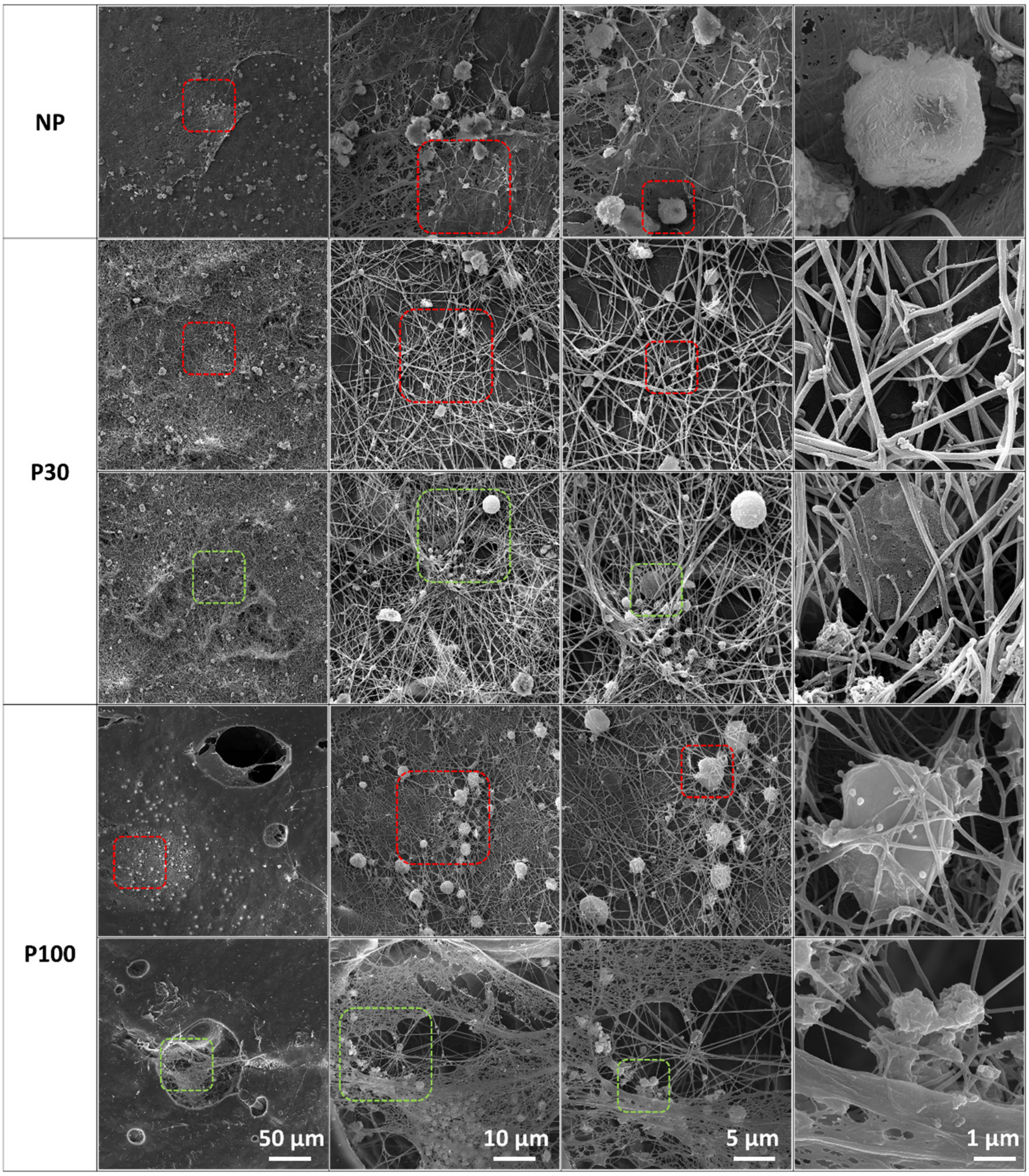

Figure S20. Whole blood clotting on PCL films. Pores on the P30 films entrap the RBCs and large pores on P100 prepare the suitable surface for blood cell attachment. The red squares show the magnified area.

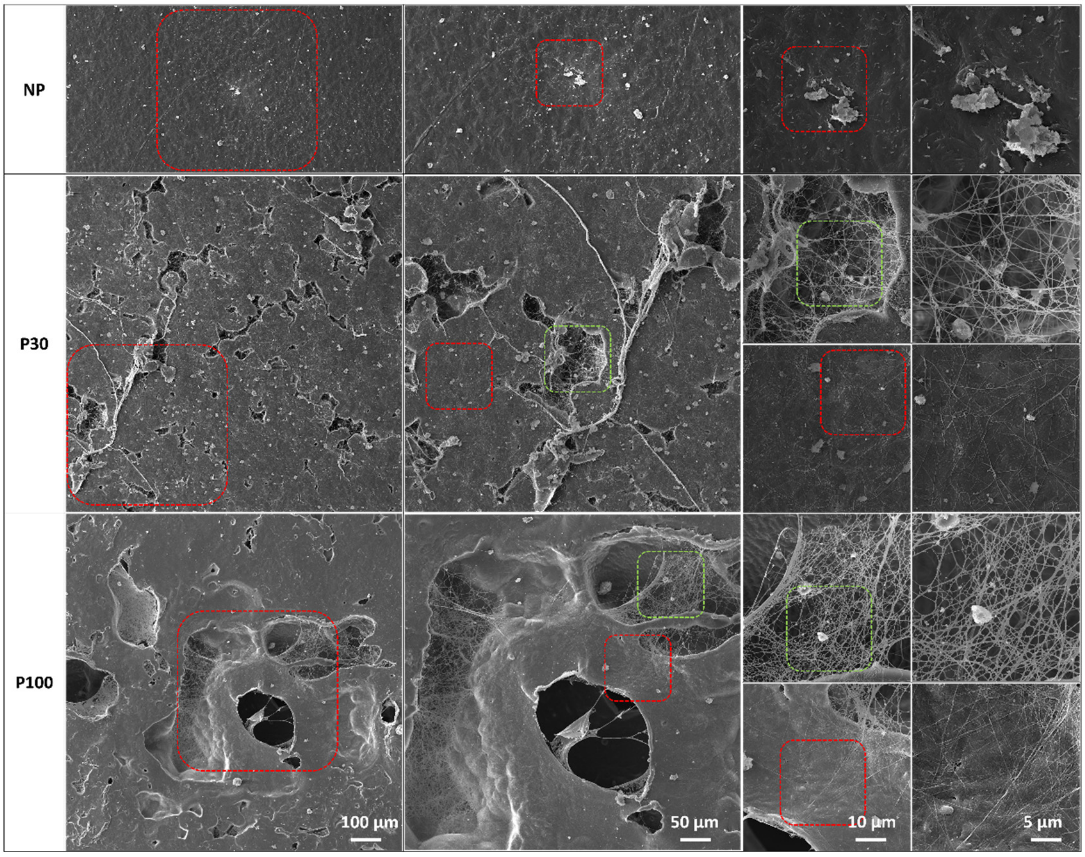

Figure S21. Plasma clotting on PCL films. Microscopic analysis shows denser fibrin network formation on porous films than the NP. The micrographs show plasma clotting on the surface and inside the pores. The red squares show the magnified area on the pores and green square show the magnified area on the surface of the films.

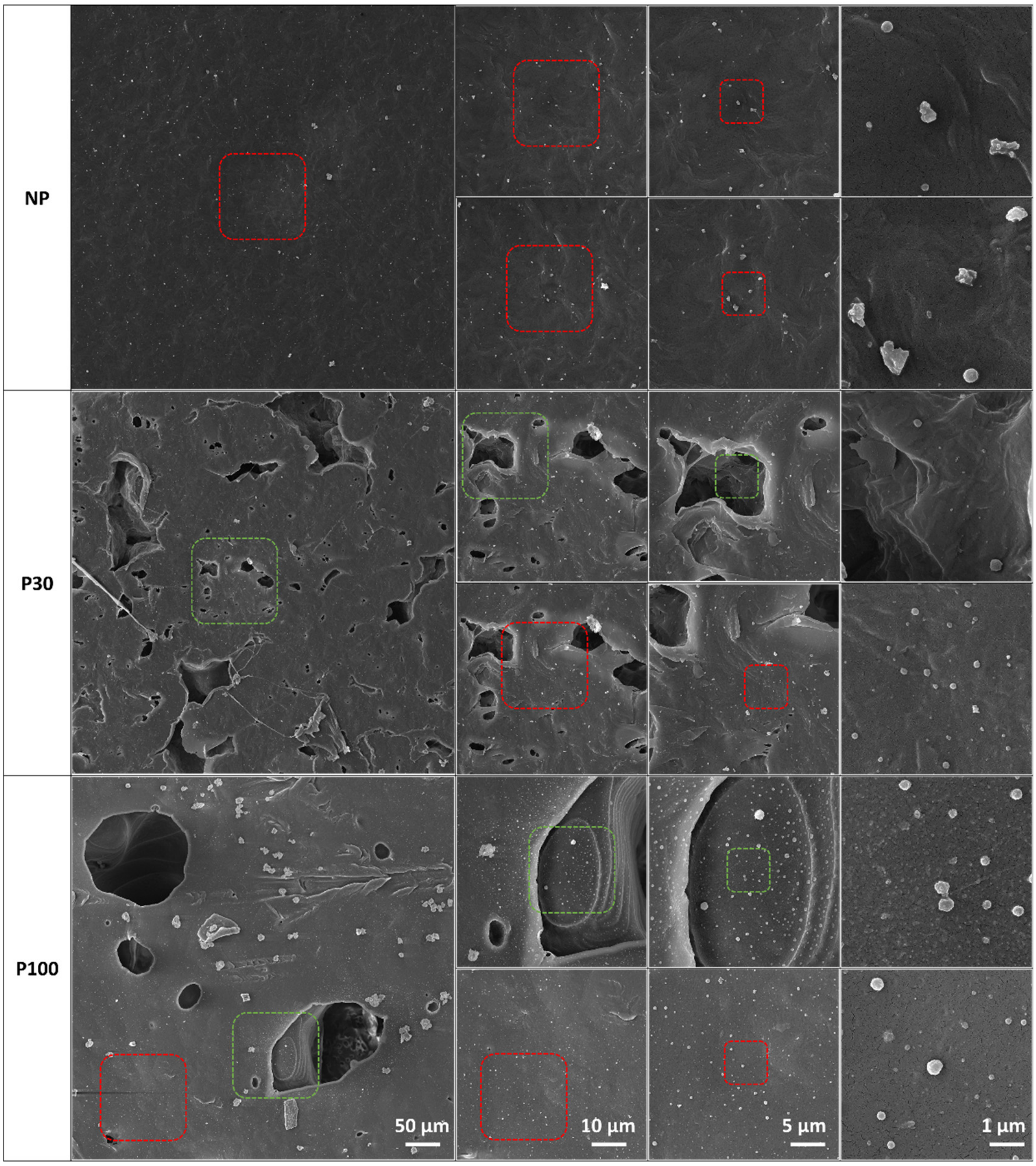

Figure S22. PRP attachment to PCL films. Microscopic analysis confirms that porous films show more PRP attachment than the NP. The micrographs show PRP attachment on the surface and inside the pores. The red squares show the magnified area.

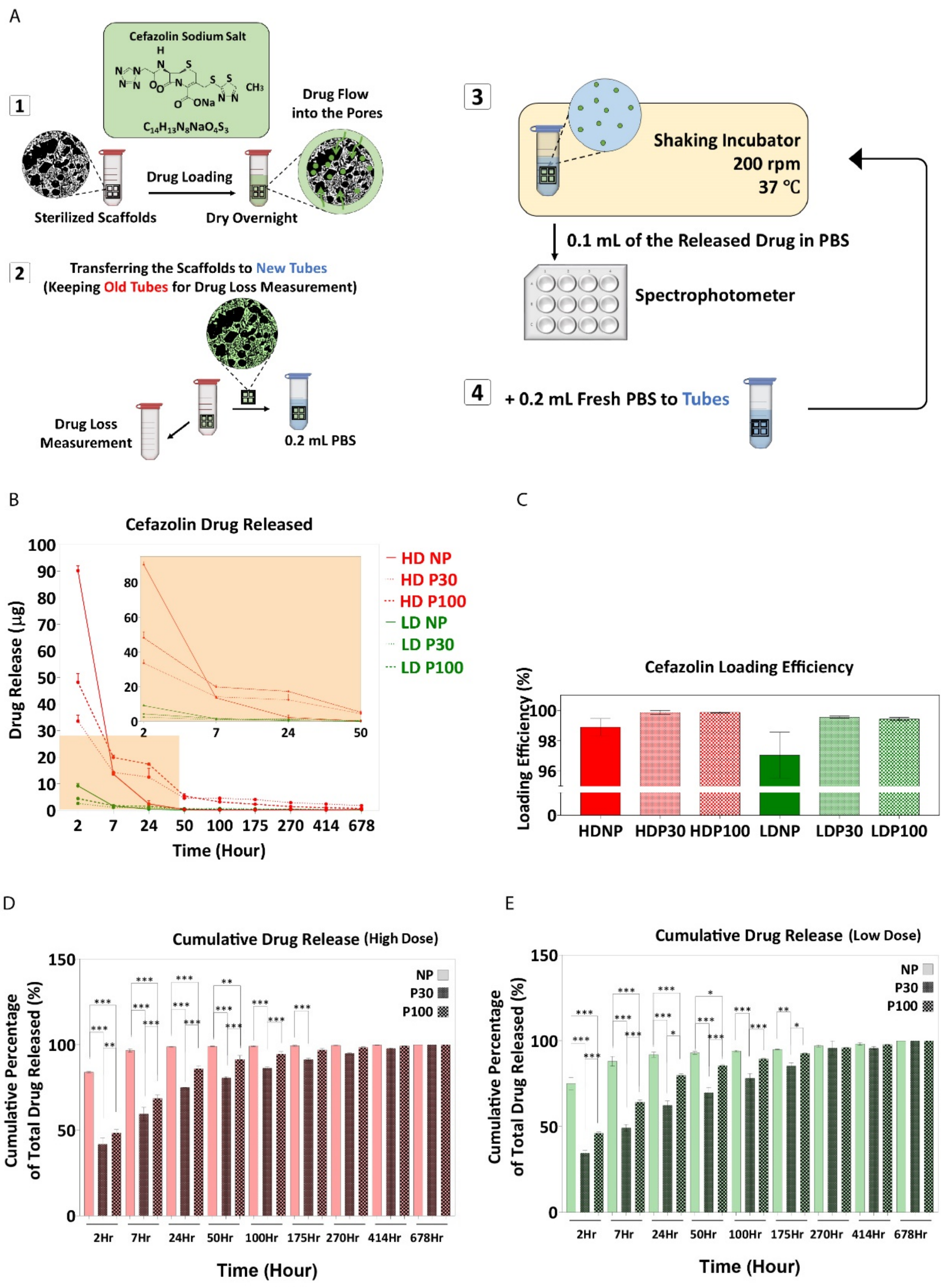

Figure S23. Cefazolin drug release. (A) Schematic presentation of the method for drug delivery experiment. (B) drug release profile and loading efficiency. (D-E) drug release analysis. *, ** and *** in figures indicate $p < 0.033$, $p < 0.002$ and $p < 0.001$, respectively.

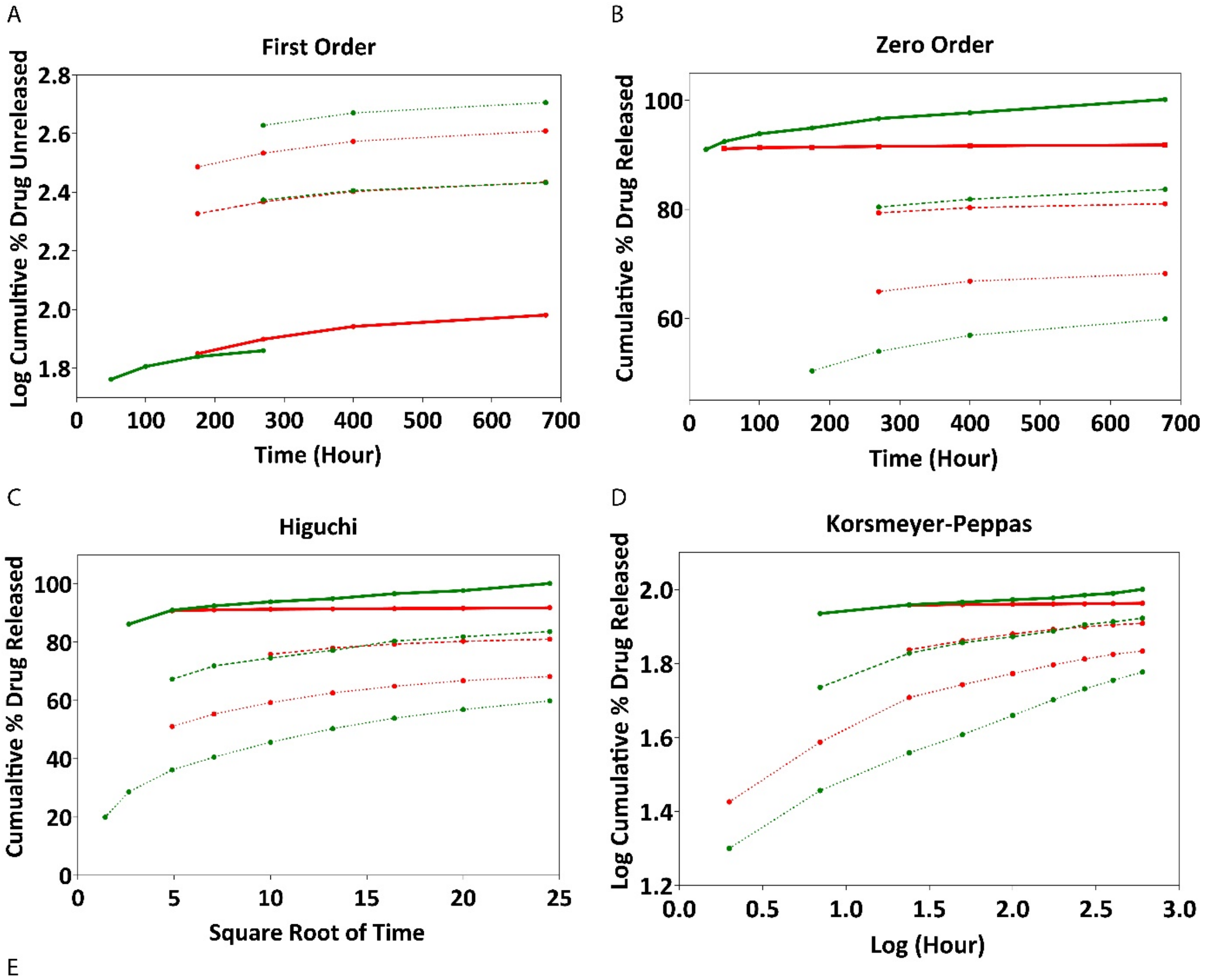

E

		Zero-Order	First-Order	Higuchi	Korsmeyer-Peppas	60 % of the release + Korsmeyer-Peppas
NP	HD	y = 0.0011x + 91.163<br>R^2 = 0.9025	y = 0.0002x + 1.8232<br>R^2 = 0.9105	y = 0.0477x + 90.703<br>R^2 = 0.931	y = 0.0033x + 1.9536<br>R^2 = 0.9876	y = 6.2629x<br>R^2 = 1
	LD	y = 0.013x + 92.056<br>R^2 = 0.9275	y = 0.0004x + 1.7528<br>R^2 = 0.9159	y = 0.5453x + 87.37<br>R^2 = 0.9148	y = 0.0306x + 1.9121<br>R^2 = 0.979	y = 6.1835x<br>R^2 = 1
P30	HD	y = 0.0076x + 63.212<br>R^2 = 0.9158	y = 0.0002x + 2.4629<br>R^2 = 0.9041	y = 0.8541x + 49.451<br>R^2 = 0.9228	y = 0.1554x + 1.4417<br>R^2 = 0.9262	y = 0.188x + 1.4072<br>R^2 = 0.9494
	LD	y = 0.0178x + 48.464<br>R^2 = 0.9181	y = 0.0002x + 2.5868<br>R^2 = 0.9374	y = 1.5929x + 25.827<br>R^2 = 0.9012	y = 0.1862x + 1.2798<br>R^2 = 0.9856	y = 0.1862x + 1.2798<br>R^2 = 0.9856
P100	HD	y = 0.0039x + 78.475<br>R^2 = 0.9184	y = 0.0002x + 2.3059<br>R^2 = 0.9089	y = 0.346x + 73.08<br>R^2 = 0.9237	y = 0.0509x + 1.7734<br>R^2 = 0.9667	y = 0.2366x + 1.5188<br>R^2 = 0.9884
	LD	y = 0.0077x + 78.502<br>R^2 = 0.9792	y = 0.0001x + 2.3419<br>R^2 = 0.9359	y = 0.8036x + 65.649<br>R^2 = 0.9394	y = 0.0898x + 1.6858<br>R^2 = 0.939	y = 0.2295x + 1.5214<br>R^2 = 0.98

Figure S24. Drug release kinetics. The graphs show how different drug doses and scaffolds follow different kinetics orders during drug release. In order to understand the mechanism and kinetics of drug

release, the results of the *in vitro* drug release study were fitted into various kinetic equations (Zero-order, First-order, Higuchi and Korsmeyer-Peppas). In the Peppas model, the mechanism of the release model is characterized by using the release exponent “n” calculated from the first 60% of the drug release. Release kinetics were fitted to the Korsmeyer-Peppas model. Nonporous scaffolds showed super case II transfer with n > 0.89 which corresponds to zero-order. All porous scaffolds showed a Quasi-Fickian diffusion mechanism with n < 0.45. Later, the release kinetic followed the Higuchi model [10-12].

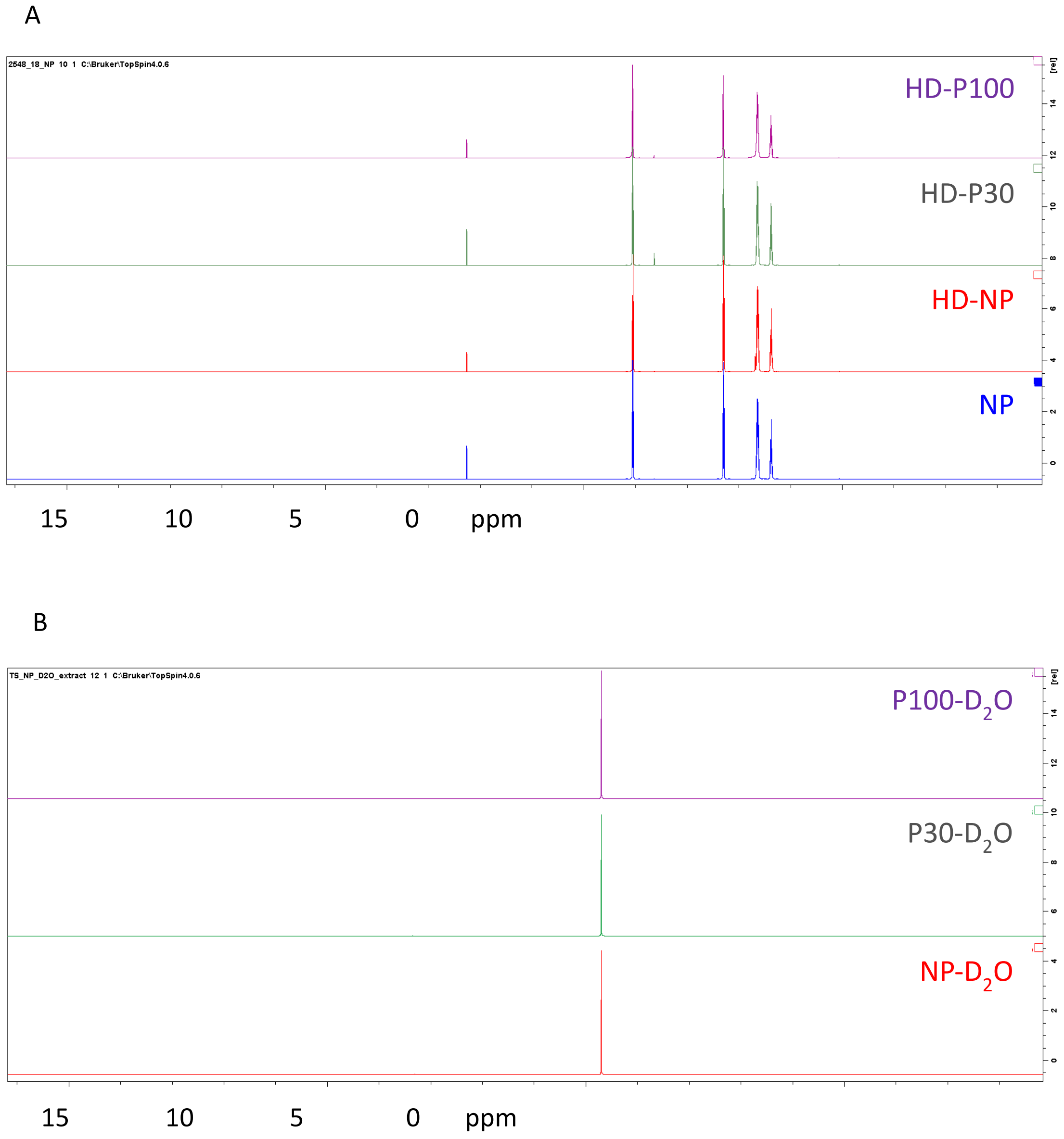

Figure S25. Cefazolin tracing inside the dug released scaffolds by ^{1}H-NMR. (A) the representative ^{1}H-NMR analysis shows there are no cefazolin spectra in the $CDCl_3$ dissolved PCL scaffolds. (B) There is no detectable cefazolin in the extracted samples which confirm the complete release of the drug. Data from NaOH cefazolin also did not show any drug release.

References

1. Domingos, M.; Dinucci, D.; Cometa, S.; Alderighi, M.; Bártolo, P. J.; Chiellini, F. Polycaprolactone Scaffolds Fabricated via Bioextrusion for Tissue Engineering Applications. *Int. J. Biomater.* **2009**, *2009*, 1–9. https://doi.org/10.1155/2009/239643.
2. Pandey, A. R.; Singh, U. S.; Momin, M.; Bhavsar, C. Chitosan: Application in Tissue Engineering and Skin Grafting. *J. Polym. Res.* **2017**, *24* (8). https://doi.org/10.1007/s10965-017-1286-4.
3. Pati, F.; Jang, J.; Lee, J. W.; Cho, D. W. *Extrusion Bioprinting*; Elsevier Inc., 2015. https://doi.org/10.1016/B978-0-12-800972-7.00007-4.
4. Zein, I.; Hutmacher, D. W.; Tan, K. C.; Teoh, S. H. Fused Deposition Modeling of Novel Scaffold Architectures for Tissue Engineering Applications. *Biomaterials* **2002**, *23* (4), 1169–1185. https://doi.org/10.1016/S0142-9612(01)00232-0.
5. Zorlutuna, P.; Annabi, N.; Camci-Unal, G.; Nikkhah, M.; Cha, J. M.; Nichol, J. W.; Manbachi, A.; Bae, H.; Chen, S.; Khademhosseini, A. Microfabricated Biomaterials for Engineering 3D Tissues. *Adv. Mater.* **2012**, *24* (14), 1782–1804. https://doi.org/10.1002/adma.201104631.
6. Raeisdasteh Hokmabad, V.; Davaran, S.; Ramazani, A.; Salehi, R. Design and Fabrication of Porous Biodegradable Scaffolds: A Strategy for Tissue Engineering. *J. Biomater. Sci. Polym. Ed.* **2017**, *28* (16), 1797–1825. https://doi.org/10.1080/09205063.2017.1354674.
7. Jakus, A. E.; Geisendorfer, N. R.; Lewis, P. L.; Shah, R. N. 3D-Printing Porosity: A New Approach to Creating Elevated Porosity Materials and Structures. *Acta Biomater.* **2018**, *72*, 94–109. https://doi.org/10.1016/j.actbio.2018.03.039.
8. Lam, C. X. F.; Savalani, M. M.; Teoh, S. H.; Hutmacher, D. W. Dynamics of in Vitro Polymer Degradation of Polycaprolactone-Based Scaffolds: Accelerated versus Simulated Physiological Conditions. *Biomed. Mater.* **2008**, *3* (3). https://doi.org/10.1088/1748-6041/3/3/034108.
9. de Luca, A. C.; Terenghi, G.; Downes, S. Chemical Surface Modification of Poly-ε-Caprolactone Improves Schwann Cell Proliferation for Peripheral Nerve Repair. *J. Tissue Eng. Regen. Med.* **2012**, 153–163. https://doi.org/10.1002/term.
10. Ofokansi, K. C.; Adikwu, M. U.; Okore, V. C. Preparation and Evaluation of Mucin-Gelatin Mucoadhesive Microspheres for Rectal Delivery of Ceftriaxone Sodium. *Drug Dev. Ind. Pharm.* **2007**, *33* (6), 691–700. https://doi.org/10.1080/03639040701360876.

11. Ofokansi, K. C.; Kenechukwu, F. C. Formulation Development and Evaluation of Drug Release Kinetics from Colon-Targeted Ibuprofen Tablets Based on Eudragit RL 100-Chitosan Interpolyelectrolyte Complexes. *ISRN Pharm.* **2013**, *2013*, 1–8. https://doi.org/10.1155/2013/838403.
12. Mircioiu, C.; Voicu, V.; Anuta, V.; Tudose, A.; Celia, C.; Paolino, D.; Fresta, M.; Sandulovici, R.; Mircioiu, I. Mathematical Modeling of Release Kinetics from Supramolecular Drug Delivery Systems. *Pharmaceutics* **2019**, *11* (3), 140. https://doi.org/10.3390/pharmaceutics11030140.
